# The More, the Merrier? Multiple Myoglobin Genes in Fish Species, Especially in Gray Bichir (*Polypterus senegalus*) and Reedfish (*Erpetoichthys calabaricus*)

**DOI:** 10.1093/gbe/evab078

**Published:** 2021-04-19

**Authors:** Kathrin Helfenrath, Markus Sauer, Michelle Kamga, Michelle Wisniewsky, Thorsten Burmester, Andrej Fabrizius

**Affiliations:** 1 Institute of Zoology, Biocenter Grindel, University of Hamburg, Germany; 2 Teaching Hospital Cologne, University of Cologne, Cologne, Germany

**Keywords:** globin, mRNA expression, sub–neofunctionalization, RNA-seq, gene expansion

## Abstract

The members of the globin superfamily are a classical model system to investigate gene evolution and their fates as well as the diversity of protein function. One of the best-known globins is myoglobin (Mb), which is mainly expressed in heart muscle and transports oxygen from the sarcolemma to the mitochondria. Most vertebrates harbor a single copy of the myoglobin gene, but some fish species have multiple myoglobin genes. Phylogenetic analyses indicate an independent emergence of multiple myoglobin genes, whereby the origin is mostly the last common ancestor of each order. By analyzing different transcriptome data sets, we found at least 15 multiple myoglobin genes in the polypterid gray bichir (*Polypterus senegalus*) and reedfish (*Erpetoichthys calabaricus*). In reedfish, the myoglobin genes are expressed in a broad range of tissues but show very different expression values. In contrast, the Mb genes of the gray bichir show a rather scattered expression pattern; only a few Mb genes were found expressed in the analyzed tissues. Both, gray bichir and reedfish possess lungs which enable them to inhabit shallow and swampy waters throughout tropical Africa with frequently fluctuating and low oxygen concentrations. The myoglobin repertoire probably reflects the molecular adaptation to these conditions. The sequence divergence, the substitution rate, and the different expression pattern of multiple myoglobin genes in gray bichir and reedfish imply different functions, probably through sub- and neofunctionalization during evolution.

Significance

Myoglobin is one of the best-known proteins in biology and usually is found as a single copy gene. In some fish species however, multiple myoglobin genes have been found but it is not well understood where these multiplications occur during fish evolution. In this study, we shed light on the independent multiplication of myoglobin genes. We found at least 15 multiple myoglobin genes in gray bichir (*Polypterus senegalus*) and reedfish (*Erpetoichthys calabaricus*) that are characterized by large sequence differences and variable gene expression. Our results indicate that myoglobin adapted rapidly during evolution in different species to similar changing abiotic environments.

## Introduction

Myoglobin (Mb) has a unique position in the history since it was the first protein with an analyzed molecular structure (Kendrew et al. 1960). Ever since these small, globular, monomeric proteins and its family members are subject to intense studies investigating the evolution and function of these proteins and genes (Graur and Li 2000; Vinogradov et al. 2007; Storz et al. 2013; Schwarze et al. 2014). Mb consists of eight alpha helices, which harbor an iron-containing heme group that enables Mb to bind gaseous ligands, such as oxygen (O_2_) and nitric oxide (NO^·^) (Wittenberg BA and Wittenberg JB 1989; Wittenberg JB and Wittenberg BA 2003). The main expression sites of Mb are the heart and skeletal. The high O_2_ affinity enables Mb to extract O_2_ of the less affine hemoglobin from the blood. Within the myocytes Mb facilitates the transport of O_2_ to mitochondria (Merx et al. 2001; Wittenberg JB and Wittenberg BA 2003; Helbo et al. 2013). Additionally, Mb stores O_2_ for short-term or long-term hypoxic stages. High concentration of Mb in muscles of diving mammals lead to a large O_2_ storage capacity, sustaining extended dives (Davis 2014). In addition to these properties, Mb also displays enzymatic functions. For example, under normoxic conditions, oxygenated Mb acts as dioxygenase, converting the bioactive NO^·^ to nitrate (${\text{NO}}_{3}^{–}$) and under hypoxic conditions deoxygenated Mb acts as a nitrite reductase producing NO^·^ from ${\text{NO}}_{2}^{–,}$ which leads to a NO^·^-mediated vasodilation and thus, enhances oxygen supply (Hendgen-Cotta et al. 2008; Hendgen-Cotta et al. 2014). It has also been assumed that *Mb* is involved in oxidative defense by detoxifying reactive oxygen species (ROS) (George and Irvine 1951; Osawa and Korzekwa 1991; Flögel et al. 2004; Helbo et al. 2012).

Surprisingly, *Mb* knockout mice show no immediate physiological defects (Garry et al. 1998). However, they exhibit multiple compensatory mechanisms, including a higher capillary density, smaller cell width, elevated hematocrit and increased coronary flow, ensuring sufficient oxygen supply (Gödecke et al. 1999; Grange et al. 2001; Meeson et al. 2001; Wittenberg JB and Wittenberg BA 2003). In addition to the occurrence of *Mb* in heart and skeletal muscles, there is recent evidence for *Mb* expression in smooth muscle, endothelial and tumor cells, even though in significantly lower levels (Qiu et al. 1998; Cossins et al. 2009; Gorr et al. 2011). In some cancer cells *Mb* is probably involved in specific cellular hypoxia response mechanisms by modulating gene expression of hypoxia marker genes, such as HIF1α (Bicker et al. 2019).

Most vertebrates harbor one *Mb* gene in the genome (Burmester and Hankeln 2014). However, there are some known exceptions that either lost *Mb* or have multiple *Mb* genes (Maeda and Fitch 1982; Fraser et al. 2006; Fuchs et al. 2006; Sidell and O'Brien 2006; Roesner et al. 2008; Hoffmann et al. 2011; Gallagher and Macqueen 2016; Koch et al. 2016; Li et al. 2018).

The stickleback and some icefish species only express nonfunctional *Mb* sequences (Hoffmann et al. 2011; Sidell and O'Brien 2006), whereas the bullfrogs (*Rana catesbeian*) completely lack the *Mb* gene (Maeda and Fitch 1982; Fuchs et al. 2006). The compensatory mechanisms are not fully understood, but it has been speculated that a hemoglobin chain in the heart of frogs adopts a Mb-like function (Maeda and Fitch 1982) and the low water temperatures in the Antarctic sea may render Mb unnecessary because the physically dissolved O_2_ in the cold water is sufficient to support the very low metabolic rate of icefish (Sidell and O'Brien 2006).

The common carp (*Cyprinus carpio*) and goldfish (*Carassius auratus*) on the other hand have two Mb isoforms (Mb1 and Mb2) with different tissue expression patterns (Fraser et al. 2006; Roesner et al. 2008). Although Mb1 displays a rather ubiquitous expression pattern (e.g., heart, liver, kidney, and gills), Mb2 is exclusively located in neuronal tissues (Fraser et al. 2006; Cossins et al. 2009). In lungfish (Dipnoi) there are up to seven distinct *Mb* genes with different kinetic properties and tissue-specific expression patterns (Koch et al. 2016; Lüdemann, Fago, et al. 2019). Interestingly, in contrast to the single copy *Mb*, which is mostly expressed in the heart, the highest expression site of lungfish Mb is the brain (Koch et al. 2016; Lüdemann, Fago, et al. 2019). The silver arowana (*Osteoglossum bicirrhosum*) and the Asian arowana (*Scleropages formosus*) harbor three *Mb* genes (Gallagher and Macqueen 2016) and the walking catfish (*Clarias batrachus*) exhibits the largest *Mb* repertoire with 15 *Mb* genes, known so far (Li et al. 2018).

We explore whether these fishes form an exceptional position or if multiple *Mb* genes are a common phenomenon within fish species. We analyzed the Sequence Read Archive (SRA) data sets (supplementary table 1, Supplementary Material online) of 12 fish species and found multiple *Mb* genes in gray bichir (*Polypterus senegalus*) and reedfish (*Erpetoichthys calabaricus*). Both species harbor at least 15 Mb genes, the largest number of *Mb* genes found so far. To better understand the evolutionary history of all described *Mb* genes, we examined the phylogeny of the novel *Mb* genes in gray bichir and reedfish. Further, we investigated the amino acid sequences and the expression pattern of the multiple *Mb* genes of gray bichir and reedfish, providing first evidences of the evolutionary fate of gene expansion in these fish. Our results give hints on why multiple *Mb* genes occur especially in these fish and provide similarities of multiple *Mb* genes in different species which hints to similar functions.

## Results

### Evolution of Multiple Mbs in Several Fish Species

**Fig. 1.**
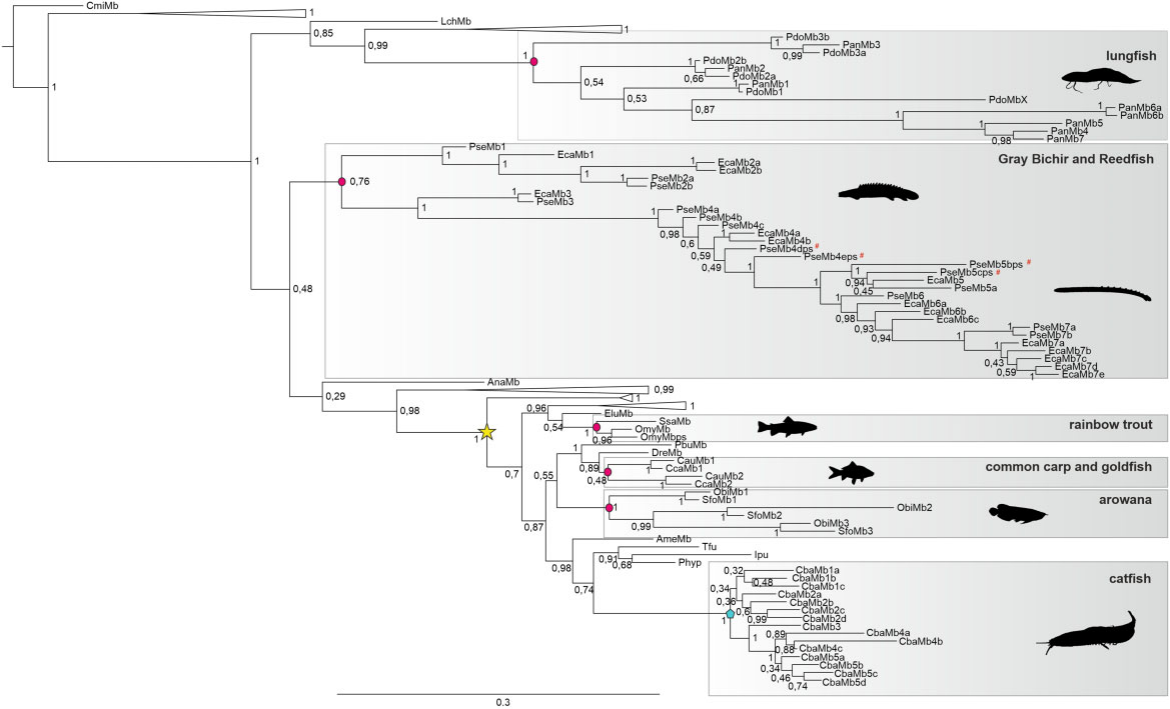
Phylogeny of multiple *Mb* genes in bony fish (Osteichthyes). The pedigree was calculated using Bayesian analysis based on the Dayhoff + I + G model. The bar represents 0.3 substitutions per amino acid position. The single copy Mb sequence of the Australian ghost shark (CmiMb) serves as the outer group. The numbers at the nodes are the Bayesian posterior probabilities. Collapsed clades are shown as triangles, the original tree without collapsed areas is shown in supplementary figure 4, Supplementary Material online, and taxa marked bold in these clades. The four pseudogenes of the gray bichir are marked with a red #. The teleost-specific whole genome duplication (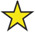) is marked, as well as the formation of multiple *Mb* genes in the last common ancestor (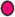) or exclusively in the species itself (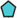).

To investigate the origin and the evolution of multiple *Mb* genes in different fish species, we generated a phylogenetic tree including all known multiple *Mb* genes (fig. 1). The single copy Mb sequence of the Australian ghost shark, a cartilaginous fish (Chondrichthyes) was used to root the tree. Some walking catfish Mb sequences were shortened since they show some incongruity (supplementary fig. 1, Supplementary Material online). *Mb* genes of lungfish, polypteridae and teleostei formed monophyletic clades, strongly indicating a distinct evolutionary divergence. Within lungfish, polypteridae, arowana and cyprinids, the multiple *Mb* genes seem to occur in the last common ancestor. This becomes apparent when some *Mb* genes of different species (that belong to one order) form sister groups, for example, *Mb2*, *Mb3*, and *Mb7* of the gray bichir and reedfish. Multiple *Mb* genes of walking catfish do not form sister groups with *Mb* genes of other catfish species. This suggests that the multiple *Mb* genes just occur in the walking catfish and are not present in the last common ancestor of catfish. Genome duplications in the evolution of salmon (Macqueen and Johnston 2014; Lien et al. 2016) presumably lead to duplicated *Mb* genes in salmon. In Rainbow trout, another salmonid fish, the duplicated *Mb* genes still exist, however, their functionality was lost due to pseudogenization. Atlantic salmon might have lost one of the duplicated Mb genes.

Polypteridae and lungfish diverged approximately 430 Ma (Kumar et al. 2017), comparison of the Mb sequences between and within these groups allows the evaluation of the amino acid evolution. The multiple *Mb* genes show different substitution rates with the lowest within the gray bichir 1.73 ± 0.17 × 10^−9^ amino acid substitutions per site per year (supplementary table 5, Supplementary Material online). In polypteridae Mb3 with 1.24 ± 0.43 × 10^−10^ amino acid substitutions per site per year shows the lowest substitution rate versus Mb1 (6.35 ± 1.18 × 10 ^−10^), Mb5 (6.62 ± 1.33 × 10 ^−10^ amino acid substitutions per site per year), and Mb7 (5.34 ± 1.15 × 10^−10^ amino acid substitutions per site per year) with up to five times higher rates, respectively (supplementary table 6, Supplementary Material online). The lower substitution rate in Mb3 could indicate a purifying selection pressure, thus conserving an important function whereas the higher substitution rates in Mb5 and Mb7 may lead to new functions for these Mb genes. Overall, the results show that the multiple Mb genes of the different fish species did not arise in one last common ancestor, rather they emerged by convergent evolution. In most cases, the multiple Mb genes arise in the family's last common ancestor and in catfish they exclusively occur in the walking catfish.

### Sequence Analysis of Multiple Mb Genes in Reedfish and Gray Bichir

**Fig. 2.**
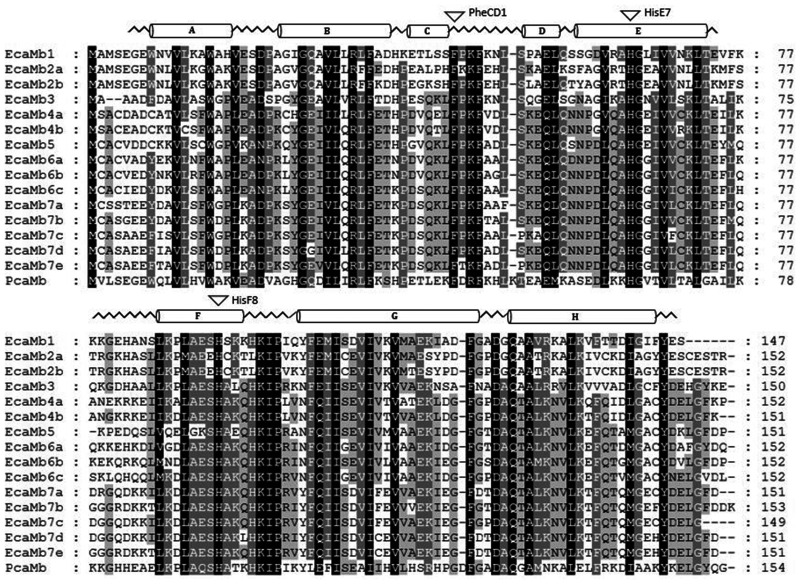
Amino acid sequence alignment of the multiple Mb genes of reedfish (*Erpetoichthys calabaricus*) and the Mb of the sperm whale (PcaMb). The secondary structure of sperm whale myoglobin is superimposed in the upper row, with α-helices designated A–H. The conserved areas are highlighted in black (100%), dark gray (75%), and gray (50%) and the conserved amino acids PheCD1, HisE7, and HisF8 are marked by arrows.

**Fig. 3.**
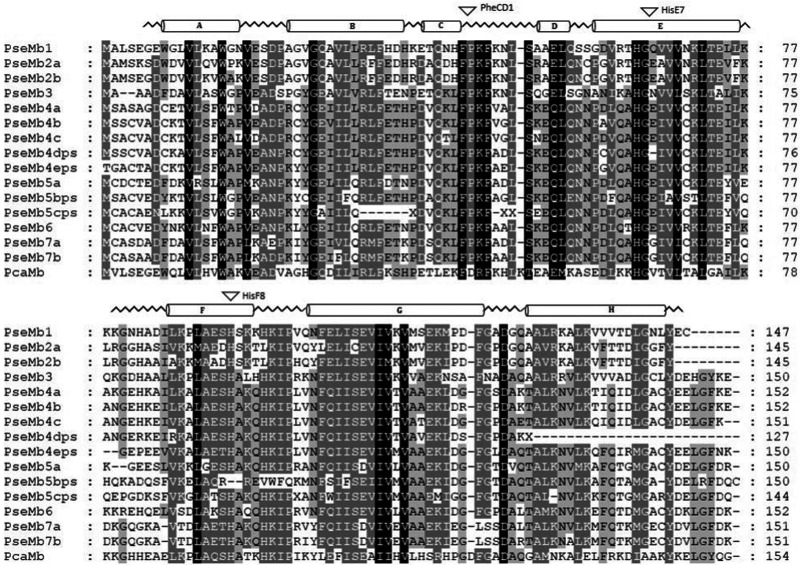
Amino acid sequence alignment of the multiple Mb genes of gray bichir (*Polypterus senegalus*) and the Mb of the sperm whale (PcaMb). The secondary structure of sperm whale myoglobin is superimposed in the upper row, with α-helices designated A–H. The conserved areas are highlighted in black (100%), dark gray (75%), and gray (50%) and the conserved amino acids PheCD1, HisE7, and HisF8 are marked by arrows. Pseudogenes are abbreviated with ps.

Sequence analysis of the newly detected multiple Mb genes in the two polypteridae species showed 15 functional Mb genes (EcaMb) in reedfish (fig. 2), whereas gray bichir (fig. 3) lost 4 of the 15 functional Mb genes (PseMb) by pseudogenization. On average, EcaMbs are composed of 151 amino acids and display identity values between 32% (EcaMb2b to EcaMb5 and EcaMb7e) and 92% (EcaMb2a and EcaMb2b). EcaMb1 showed the highest identity to the single copy Sperm whale PcaMb by sharing 48% of amino acids, whereas EcaMb5 displayed the biggest differences to the single copy PcaMb (32%). Amino acids that are essential for oxygen binding including the distal and proximal histidines at helix positions E7 and F8 and the phenylalanine residue at position CD1 are present in all EcaMb sequences (fig. 2).

PseMb genes consist of 148 amino acids on average. PseMb2a and PseMb2b showed the highest identity with 92%, whereas PseMb1 and PseMb5bps differ the most (33%). Similar to EcaMbs, PseMb1 exhibited the highest identity value with the single copy PcaMb with 47%, whereas PseMb5bps only share 27% of amino acids with PcaMb. During evolution 4 of the 15 PseMb genes became pseudogenes and thus, lost their function. In PseMb4dps and PseMb5cps a stop codon prematurely interrupts their CDS, PseMb4e lost the start codon and PseMb5bps lost the histidine at helix positions F8 (fig. 3). In summary, the sequence analysis indicates an expansion of Mb genes by amplification in reedfish and reduction by pseudogenization in gray bichir.

### Gene Expression of Multiple Mb Genes in Reedfish and Gray Bichir

Gene expression analysis with EcaMb and PseMb were performed on 10 tissues by using qRT-PCR and on different tissues and mixtures by analyzing freely available SRA transcriptome data sets. Due to high similarity of some Mb genes it was not possible to clone each Mb and thus, we could not perform the expression analyses for three EcaMbs and two PseMbs via qRT-PCR.

**Fig. 4.**
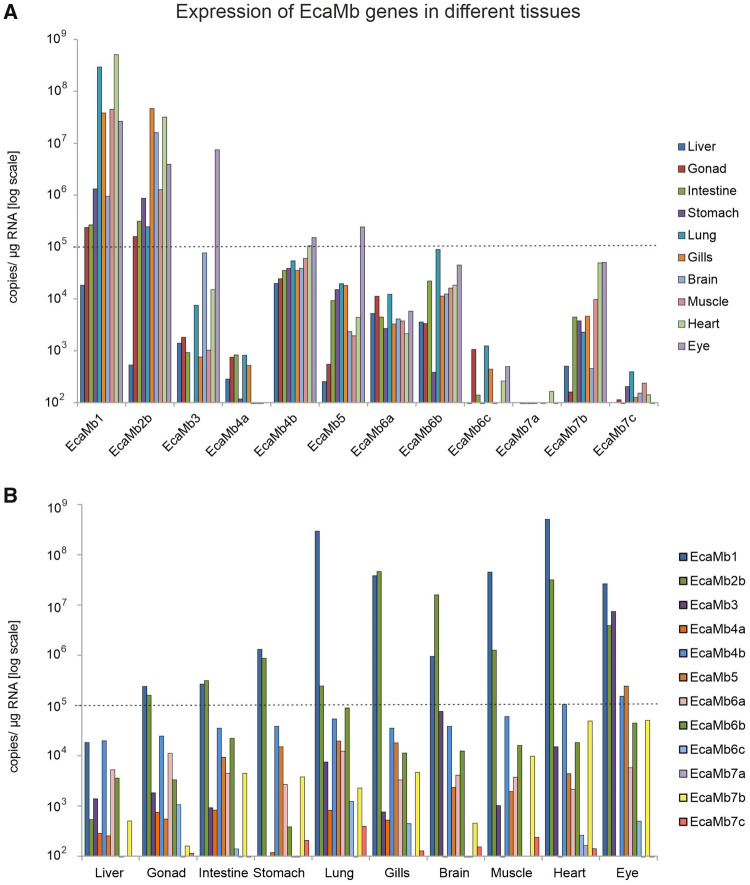
Gene expression analysis of the multiple Mb genes in different tissues of reedfish employing the qRT-PCR. Gene expression values are shown as absolute copy number per µg RNA. Expression values above 10^5^ copies/µg RNA are marked by the dashed line. (*A*) Tissue-specific gene expression of EcaMbs. (*B*) Expression of EcaMbs sorted per tissue.

Most of the EcaMb genes exhibited a rather ubiquitous expression pattern determined by qRT-PCR (fig. 4). However, the gene expression levels of EcaMbs clearly showed different intensities. EcaMb1, EcaMb2b, and EcaMb3 showed the highest expression; whereas EcaMb1 was most highly expressed in heart (5.09 × 10^8^ copies per µg total RNA), lungs (2.94 × 10^8^ copies per µg total RNA), muscles (4.50 × 10^7^ copies per µg total RNA), gills (3.83 × 10^7^ copies per µg total RNA), and eye (2.63 × 10^7^ copies per µg total RNA); EcaMb2b was found in gills (4.66 × 10^7^ copies per µg total RNA), heart (3.18 × 10^7^ copies per µg total RNA), and brain (1.59 × 10^7^ copies per µg total RNA), EcaMb3 was found to be expressed with high levels in the eye (7.46 × 10^6^ copies per µg total RNA). In addition, EcaMb4b and EcaMb5 showed expression values above 10^5^ copies per 1 µ RNA in eye. Thus, the eye in reedfish showed the strongest expression and the largest number of EcaMb genes, whereas the liver showed the lowest expression values (fig. 4*B*). Liver and gill SRA-transcriptome analyses are in line with our qRT-PCR findings (supplementary fig. 2*A* and *B*, Supplementary Material online). Surprisingly, EcaMb4a and EcaMb4b showed the highest expression values in the mixture of gills, brain, liver and ovary (supplementary fig. 2*C*, Supplementary Material online). Since we found moderately high expression levels in gills, brain and liver via qRT-PCR but did not analyze the ovary, it may indicate very high EcaMb4a and EcaMb4b expression in ovary. The low expression values of EcaMb1, EcaMb2b and EcaMb3 in mixture of gills, brain, liver and ovary are not in accordance with our qRT-PCR results. That could be due to a bioinformatical artifact or faulty mapping based on very similar transcripts/reads.

**Fig. 5.**
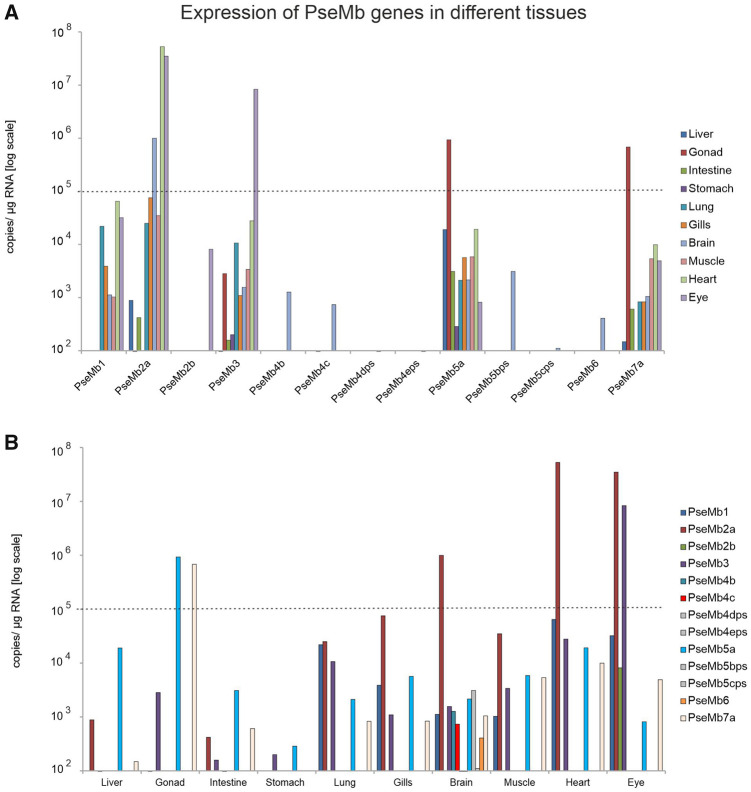
Gene expression analyses of the multiple Mb genes in different tissues of gray bichir. Expression values above 10^5^ copies/µg RNA are marked by the dashed line. (*A*) Tissue-specific gene expression of PseMbs. (*B*) Expression of PseMb sorted per tissue.

In contrast to EcaMb genes, the expression of PseMb genes is rather scattered throughout the tissues we analyzed by qRT-PCR (fig. 5). PseMb1, PseMb2a, PseMb3, PseMb5, and PseMb7a show expression values of above 10^5^ copies per µg RNA, whereas PseMb2a showed its highest expression in heart (5.28 × 10^7^ copies per µg total RNA), eyes (3.50 × 10^7^ copies per µg total RNA), and brain (9.98 × 10^5^ copies per µg total RNA). PseMb5a and PseMb7a showed a clear expression maximum in gonads (PseMb5a 9.32 × 10^5^ copies per µg total RNA; PseMb7a 6.81 × 10^5^ copies per µg total RNA), whereas PseMb3, similar to the EcaMb3, was most highly expressed in the eye (8.37 × 10^6^ copies per µg total RNA). Thus, there are two PseMb genes in gonads and in eye with a copy-number higher than 10^5^ copies per 1 µg RNA. Interestingly, almost all PseMb genes are expressed in the brain. However, the expression values are minor with the exception of PseMb2a (9.98 × 10^5^ copies per µg total RNA). SRA-transcriptome analysis confirmed the high expression values of PseMb1 in developing tissue of mandible and in pectoral fin (supplementary fig. 3*B* and *C*, Supplementary Material online). Additionally, the high expression of PseMb6, PseMb7a, and PseMb7b found in a mixture of heart, liver, spleen, kidney and brain (supplementary fig. 3*A*, Supplementary Material online) may indicate a strong expression in spleen and kidney.

## Discussion

Although most vertebrates harbor a single copy Mb gene, some fish species evolved multiple Mb genes. This multitude of Mb genes is probably of advantage for these species. We were interested how these multiple Mb genes have evolved and whether this phenomenon is unique to only few species or rather common in teleost fish. Our results show a convergent evolution of multiple Mb genes in which the genes mostly occur in the last common ancestor of each order. Further we found at least 15 Mb copies in gray bichir and reedfish with different expression patterns and various sequence divergences.

### Multiple Mb Genes Occurred Independently in Different Fish Species, but Seem to Have Arisen for Similar Reasons

Multiple Mb genes do not occur in the last common ancestor of Osteichthyes but rather originated at least six times convergently as shown in our results (fig. 1). With the exception of the walking catfish, the multiple Mb genes occur in the last common ancestor of each order. Some of the multiple Mb genes show high substitution rates, in particular the Mbs in lungfish and some Mbs in gray bichir and reedfish. Fast changes in the CDS may be due to the change of selection pressures after functional diversification and changes in tissue expression (Helbo et al. 2012).

The substitution rates of the walking catfish Mb genes are unclear since we have shortened some of the sequences due to incongruity. The multiple Mb genes of the walking catfish were only found in this species and do not occur in other catfish species. The exact evolutionary origin of walking catfish Mb genes is not fully understood, but genome sequence analyses show that the Mb genes presumably multiplied by tandem duplication (Li et al. 2018).

We found at least 15 Mb genes in gray bichir (*P. senegalus*) and reedfish (*E. calabaricus*), dating the multiplication to the last common ancestor approximately 400 (Ma), similar to lungfish Mb genes that also occur in the last common ancestor approximately 400 Ma (Betancur-R et al. 2013; Berthelot et al. 2014). Polypteridae and lungfish diverged 430 Ma, which indicates a strong abiotic influence in this interim period and could have favored the emergence of multiple Mb genes. The oxygen concentration started to rise 440–300 Ma from approximately 18% to approximately 30%, which might have played a major role in the emergence of multiple Mb genes (dates from http://www.timetree.org; last accessed September 28, 2020 [Kumar et al. 2017]).

Some lungfish species exhibit the largest genomes of animals with *C*-values of 40 pg up to 133 pg. The genome of polypteridae (3.7–7.3 pg) is much smaller compared with lungfish. Nevertheless, the genome of polypteridae is larger than genomes of carp (1.8 pg), zebrafish (1.8 pg), and human (3.5 pg) (Gregory 2020). Even though the lungfish have the largest genome, there is no evidence for particular polyploidization (Vervoort 1980) and thus, there is no correlation between multiple Mb genes with the size of the genome (Koch et al. 2016). The multiple Mb genes of lungfish and probably also of gray bichir and reedfish are outcomes of Mb-specific gene multiplication events.

Although the multiple Mb genes occurred independently in different fish species, they seem to have evolved for similar reasons. In their environment, fish are exposed to fluctuating oxygen concentrations, which can lead to hypoxia and oxidative stress (Nikinmaa 2002; Nikinmaa and Rees 2005; Tiedke et al. 2014; Missaghi et al. 2017). Interestingly, fish with multiple Mb genes are extremely well adapted to this dynamic environment. For example, the Goldfish is able to survive anoxic periods (Lushchak et al. 2001), the lungfish, the gray bichir, and the reedfish exhibit lungs whereas the walking catfish possesses suprabranchial chambers, which enable them to breath atmospheric air (Jesse et al. 1967; Lechleuthner et al. 1989; Lefevre et al. 2014). Further, lungfish and the walking catfish aestivate in a mud cocoon during the hot summer periods, whereas gray bichir, reedfish and also the walking catfish survive dry periods by crossing terrestrial areas to find water sources (Delaney et al. 1974; Bruton 1979; Pace and Gibb 2011; Standen et al. 2014). These morphologic and behavioral adaptations enable these fish species to cope with changing oxygen concentrations. However, adaptations on the molecular level are also needed to prevent cell damage as a result of hypoxia and reoxygenation.

Presumably the multiple Mb genes play a role in this molecular adaptation and have a cytoprotective role in the organism. Although Mb1 in goldfish and common carp displays a ubiquitous expression pattern and seem to be involved in oxygen supply and NO-metabolism, Mb2 is restricted to the brain and protects it by detoxifying ROS (Fraser et al. 2006; Roesner et al. 2008; Cossins et al. 2009; Helbo et al. 2012). High expression values of Mb genes in the brain were also found in some lungfish and the walking catfish. Lungfish Mbs protect cells from hypoxia and reduce the production of ROS (Koch et al. 2016). Mb genes of gray bichir and reedfish also show high expression values in brain, but also in other tissues. Presumably, some of the multiple Mb genes protect tissues sensitive to hypoxia and oxidative stress, and other Mb genes that are expressed in respiratory tissues might be involved in oxygen supply. Investigations of multiple Mb genes in goldfish and lungfish show that they have cytoprotective properties and play a role in the molecular adaptation to fluctuating oxygen concentrations. Like lungfish and goldfish, Mb gene in the polypteridae shows a wide range of expression patterns, with Mb genes also being expressed in the brain and thus, might have similar functional roles.

### Evolution and Gene Expression Analyses of Multiple Mb Genes in Gray Bichir and Reedfish Suggest Functional Diversity

To obtain first insights into the evolutionary fate of multiple Mb genes in reedfish and gray bichir we investigate the phylogeny, the amino acid sequences and gene expression patterns. Our results show that Mb1 to Mb3 of both species exhibit comparatively low substitution rates and high expression values in heart. Further, Mb1 is most similar to the single copy Mb gene of sperm whale (figs. 1–5). Thus, these genes have changed little and have retained their original place of expression (Opazo et al. 2015). Probably some of these multiple Mb genes (Mb1–Mb3) have taken over the function of the original Mb gene via subfunctionalization.

Further, there are similarly high sequence differences of multiple Mb genes within gray bichir (67%) and reedfish (68%) compared with lungfish (67.6%). The high sequence divergences of lungfish Mb genes indicate different functions of these genes (Koch et al. 2016). Likely, this also applies to the multiple Mb genes in the gray bichir and reedfish. Especially coding sequences (CDs) of Mb5, Mb6 and Mb7 in gray bichir and reedfish changed extremely, which is shown by high substitution rates (fig. 1) and a high divergence to the single copy Mb gene of sperm whale (figs. 2 and 3). Interestingly, PseMb5a and PseMb7a show high expression values in gonads similar to the high expression levels of Mb5 in gonads of the west African lungfish and Mb3 in south American lungfish (Koch et al. 2016; Lüdemann, Fago, et al. 2019). Further, very high expression values of Globin E (GbE) have been described in ovaries of south American lungfish (*Lepidosiren paradoxa*, Lpa). Kinetic features of multiple LpaGbE genes show that these genes probably act as oxygen transport proteins and are involved in the NO^–^ metabolism in the south American lungfish (Lüdemann, Verissimo, et al. 2019). Thus, it is feasible that the PseMb genes fulfill similar functions in the gray bichir, protecting the sensible gonads against an imbalance in NO^-^ metabolism and insufficient oxygen.

Highest expression values of PseMb genes were measured in the heart (PseMb2a 5.2 × 10^7^ copies/µg RNA) and in the eye (PseMb2a 3.5 × 10^7^ copies/µg RNA, PseMb3 8.3 × 10^6^ copies/µg RNA). As shown in a range of vertebrate species, the heart is the tissue in which most of the Mbs are expressed (Wittenberg JB and Wittenberg BA 2003). Within the heart, Mb transports oxygen from the sarcolemma to the mitochondria of heart cells (Wittenberg JB and Wittenberg BA 2003) and also exhibits enzymatic functions by converting nitric oxide (NO^·^) into nitrate (${\text{NO}}_{3}^{–}$) under normoxic conditions (Flögel et al. 2010). High amounts of Mb protein in the cell can lead to aggregation depending on the net surface charges of the Mbs (Mirceta et al. 2013; Berenbrink 2021). The differences in the amino acid sequences we found in the gray bichir and reedfish (figs. 2 and 3) could alter the folding stabilities of the different Mbs. The changes in amino acids, depending on the position of the residues within the folded protein, may also change the net surface charge *Z*_Mb_ of the different proteins in the same tissue and add to electrostatic repulsion between Mbs within the same cell. Further analysis of the *Z*_Mb_ can shed some light on the possible distinct functions and interactions of the multiple Mbs.

PseMb2a may fulfill these functions in the heart of gray bichir. Interestingly, PseMb1 seem to have lost this classical function, since it shows very low expression values, although it is the most conserved PseMb gene and it has the highest similarity to the single copy Mb of sperm whale (fig. 3). We suppose that as a consequence of the gene duplication, the sequence of PseMb2a was able to adapt optimally to this function, whereupon this function was transferred from PseMb1 to PseMb2a. Furthermore, PseMb2a and PseMb3 display high expression values in the eye. This high expression is somehow similar to GbE expression in the eye of birds, where GbE supplies oxygen to the metabolic active retina (Blank et al. 2011). Phylogenetic analyses and O_2_ binding characteristics indicate that Mb and GbE can perform the same function (Burmester and Hankeln 2014). Thus, it is possible that PseMb2a and PseMb3 supply oxygen in the eye of gray bichir, similar to GbE in birds.

Compared with the expression pattern of PseMbs, the multiple Mb genes of reedfish showed a rather ubiquitous expression distribution. Highest expression values of EcaMb1, EcaMb2b, and EcaMb3 were measured in lungs, gills, brain, muscles, heart, and eye. Within the brain it is possible that some of the multiple EcaMbs act similar to lungfish Mbs and supply oxygen to the brain and protect it against oxidative stress. Compared with PseMbs, the most conserved EcaMb1 shows the highest expression in the heart and presumably fulfills the classical oxygen transport function in heart cells. The eye exhibits the most highly expressed EcaMb genes (EcaMb1 2.6 × 10^7^ copies/µg RNA, EcaMb2b 3.9 × 10^6^ copies/µg RNA, EcaMb3 7.4 × 10^6^ copies/µg RNA) which may indicate oxygen transport function similar to GbE in bird (Blank et al. 2011). High expression values in lung and gills of EcaMbs may indicate that these Mb genes are able to bind oxygen on respiratory surfaces. In order to determine and confirm the actual functions, further investigations must be carried out.

## Conclusion

In the present study, we show with gray bichir and reedfish that there are two more fish species with multiple Mb genes. Our results support the convergent evolution of multiple Mb genes in different species. However, they seem to have evolved for similar reasons. The gray bichir and the reedfish exhibit at least 15 Mb genes with a high sequence divergence and distinct expression patterns, indicating different functions and thus, supporting the sub- and/or neofunctionalization model in duplicated genes. The presence of multiple Mb genes demonstrates that the globin family served repeatedly during evolution to allow organisms, such as fish, to dynamically adapt to changing abiotic environments. Thus, globin genes with a large functional repertoire are a classic example to study the gene fate in terms of sub- and neofunctionalization during evolution.

## Materials and Methods

### Identification of Novel Mb-Transcripts

To identify multiple Mb genes, we investigated SRA data sets of 12 fish species available at NCBI (supplementary table 1, Supplementary Material online) including gray bichir (*P. senegalus*) and reedfish (*E. calabaricus*).

At first, de-novo assemblies were generated with Trinity (Grabherr et al. 2011). Known globin sequences were used to search for globins using the BLAST algorithm by employing GhostX (Suzuki et al. 2014). The sequences of these contigs were loaded into the *open reading frame finder* (https://www.ncbi.nlm.nih.gov/orffinder, last accessed November 04, 2020) to determine the CDS. To confirm the CDS, we performed a back-mapping with CLC-Genomics Workbench (version 11.0.1, Qiagen) and determined the exact Mb sequences of gray bichir and reedfish (supplementary tables 2 and 3, Supplementary Material online). Mb sequences of the gray bichir that lack an open reading frame, either due to frameshift or a point mutation leading to a premature stop codon, were designated as pseudogenes.

### Phylogenetic Analyses

Sequence alignment of Mb amino acid sequences of all fish that were included into the tree was created by employing MAFFT online tool with the auto strategy. The α-helices (helix A–H) are shown above the alignment (figs. 2 and 3), and were obtained using the Mb single copy sequence of the sperm whale (*Physeter catodon*, Pca). To investigate the phylogenetic relationships of multiple Mb genes in bony fish (Osteichthyes), especially the classification of polypteridae (Polypteriformes) Mbs, a multiple sequence alignment of the amino acid sequences was created with the MAFFT online tool (Katoh et al. 2009). Multiple Mb sequences of gray bichir (*P senegalus*), reedfish (*E. calabaricus*), west African lungfish (*Protopterus annectens*) (Koch et al. 2016), common carp (*C. carpio*), goldfish (*C. auratus*) (Fraser et al. 2006; Roesner et al. 2008), silver arowana (*O. bicirrhosum*), Asian arowana (*S. formosus*) (Gallagher and Macqueen 2016), and walking catfish (*C. batrachus*) (Li et al. 2018) were included in a collection of single copy Mb sequences (supplementary table 4, Supplementary Material online). The phylogenetic tree was calculated with MrBayes v3.2.2 using the Dayhoff+I + G model, which was selected using PROTTEST (Darriba et al. 2011). Two independent runs of 5,000,000 generations with four simultaneous chains each were performed. Every 1000th generation, the tree was sampled and the posterior probabilities were estimated after discarding the initial 25% of the trees.

To calculate amino acid substitution rates first the gamma distribution was estimated using the Dayhoff et al. (1978) model (+Gamma +Invar +Freq) (Schwartz and Dayhoff 1979). The estimated value of the shape parameter (2.4848) was used to calculate the evolutionary rates within and between the three groups of Mb sequences of the lungfish, reedfish, and gray bichir (supplementary table 5, Supplementary Material online). Additionally, the evolutionary divergence between selected pairs of Mbs was calculated (supplementary table 6, Supplementary Material online). Evolutionary analyses were conducted in MEGA X (Kumar et al. 2018).

### Tissue Preparation and RNA Extraction

One specimen of gray bichir and one of reedfish were obtained from a pet shop and euthanized in icy water supplemented with 1 g/l tricaine methanesulfonate. Animal handling was conducted according to the German Animal Welfare Act. Selected tissues were removed, transferred to RNAlater (Qiagen, Hilden, Germany) and stored at –80 °C until further use.

Total RNA was extracted from selected tissues using peqGOLD Trifast (PEQLAB, Erlangen, Germany) and the Crystal RNA Mini Kit (Biolab Products, Gödenstorf, Germany) following the instructions of the manufacturer, including an on-column digestion with RNase-free DNase (Qiagen, Hilden, Germany). The RNA concentration was measured with the Nanodrop ND 1000 UV-Vis spectrometer (Thermo Scientific, Bonn, Germany) and the quality and integrity of the total RNA was evaluated employing the Tapestation System (Agilent 4200). In total 1 µg RNA was used for cDNA synthesis using the RevertAid H Minus First Strand cDNA Synthesis Kit (Thermo Scientific, Bonn, Germany) following the instructions of the manufacturer. The cDNA was used for cDNA cloning and qRT-PCR.

### cDNA Cloning

To establish standard plasmids for qRT-PCR individual Mb-Sequences were amplified with gene-specific oligonucleotides that were created using the bioinformatically determined sequences. The amplicons were cloned into the pGEM-T vector (Promega, Mannheim, Germany) and sequenced by a commercial service (GATC, Konstanz, Germany).

### Quantitative Real-Time Reverse Transcription PCR

Mb mRNA expression in different tissues of gray bichir and reedfish was estimated by quantitative real-time PCR (qRT-PCR). qRT-PCR was carried out on an ABI 7500 Real-Time PCR System (Applied Biosystems, Darmstadt, Germany) and the reactions were performed in duplicates of 10 µl including the Power SYBR-Green PCR Master Mix (Thermo Scientific, Bonn, Germany). We used 1 µl of cDNA and 0.3 µl forward as well as 0.3 µl reverse primers (stock solution 10 µM). Amplification was carried out using a standard PCR protocol (95 °C 10 min, 95 °C 15 s, 60 °C 15 s, 72 °C 30 s, 40 cycles) and specific primer combinations to amplify each of the multiple Mb sequences. To validate the specificity of each amplification reaction, a dissociation curve was performed (95 °C 15 s, 60 °C 30 s, 2 cycles). We employed the standard curve method to calculate the absolute mRNA copy numbers by using serial dilutions (from 10^7^ to 10^3^ copies) of the recombinant plasmid. Standard curve reactions were run as triplicates.

### Expression Analysis via RNA-seq

In addition to the qRT-PCR analysis, we examined the gene expression of the multiple Mb-genes via RNA-Seq analysis of different organs in adult and developing specimens by using free available NCBI data sets (supplementary table 1, Supplementary Material online). Only reads with a similarity of 95% to the reference sequence in at least 95% of their length were used for further analysis. The data were normalized according to the size of the data set and the transcript length. Expression levels were calculated as reads per kilobase of transcript length per million reads (RPKM).

## Supplementary Material

Supplementary data are available at *Genome Biology and Evolution* online.

## Supplementary Material

evab078_Supplementary_Data
