# Supplementary material for: The More, the Merrier? Multiple Myoglobin Genes in Fish Species, Especially in Gray Bichir (Polypterus senegalus) and Reedfish (Erpetoichthys calabaricus)
Source: Genome Biol Evol. 2021 Apr 19;13(7):evab078. doi: 10.1093/gbe/evab078 (PMC8480196; doi:10.1093/gbe/evab078)
Supplement: evab078_Supplementary_Data [file evab078_Supplementary_Data.docx]

Supporting Information


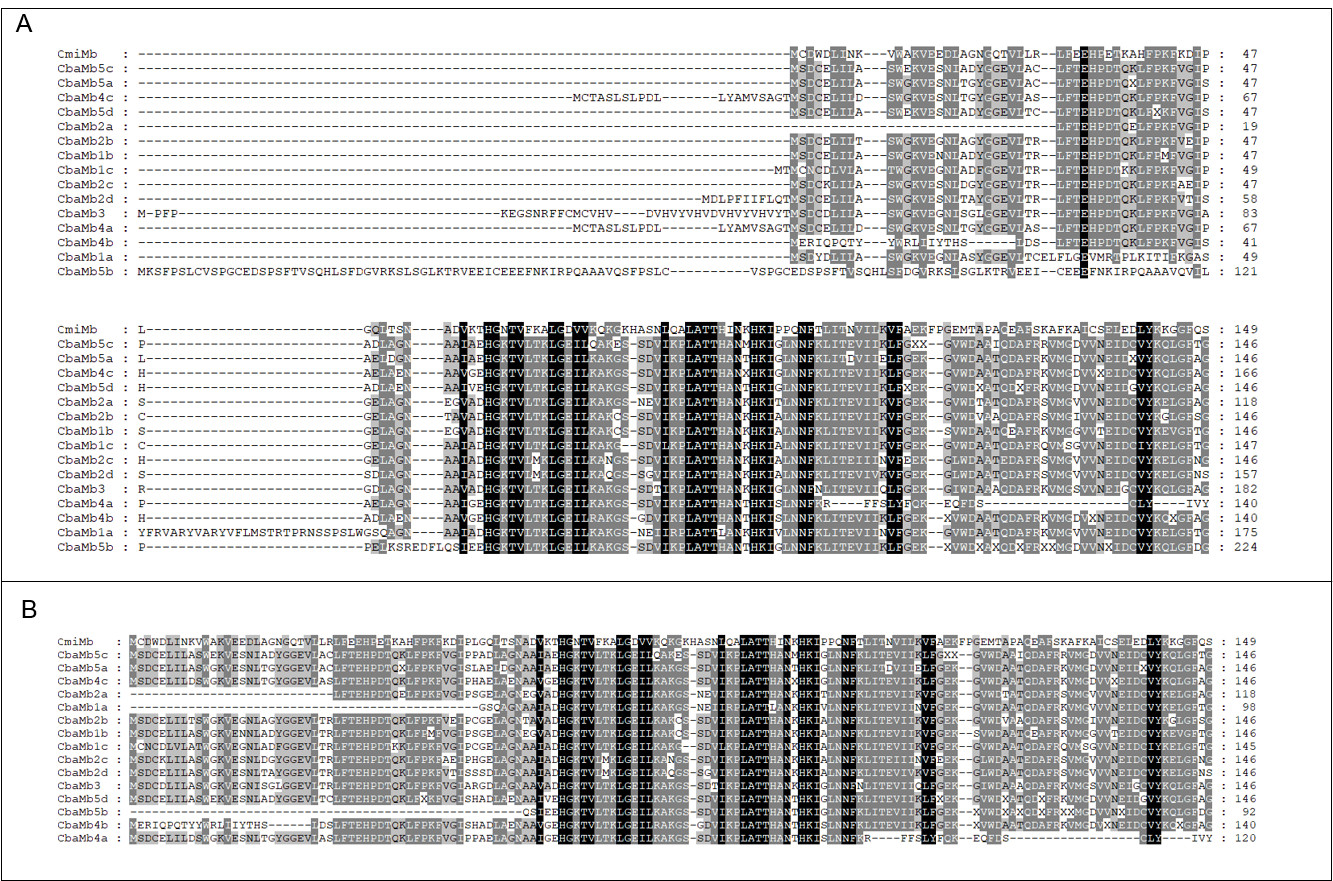


Supplementary Figure 1

(A) the complete Mb sequences of walking catfish (Cba1a – Cba5d) aligned with the single copy Mb gene of the Australian ghost shark. (B) The trimmed Mb sequences of walking catfish.


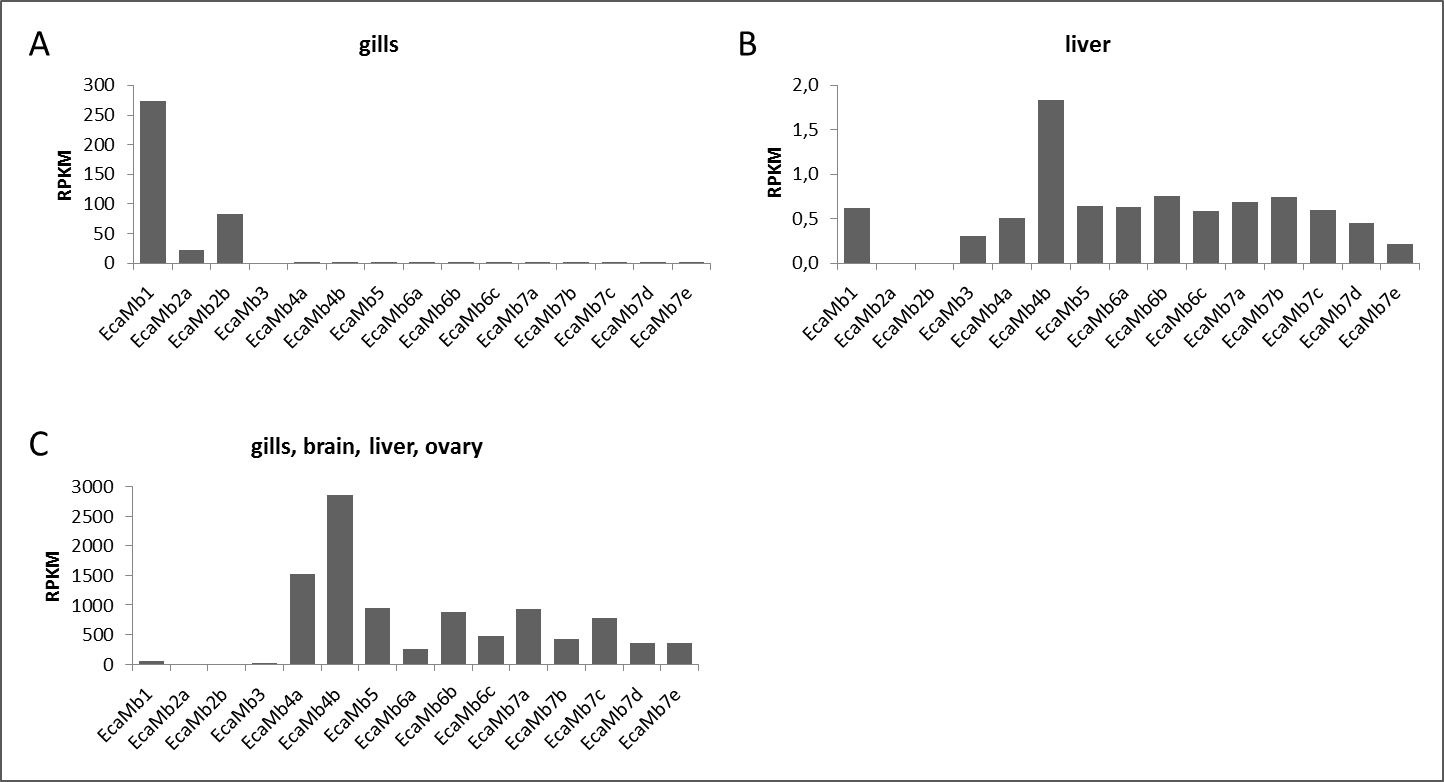


Supplementary Figure 2

Gene expression of reedfish Mbs were estimated by RNA-Seq and are displayed as RPKM values. (A) Expression of EcaMbs in gills, (B) expression of EcaMbs in liver and (C) Expression in a mixture of gills, brain, liver and ovary.


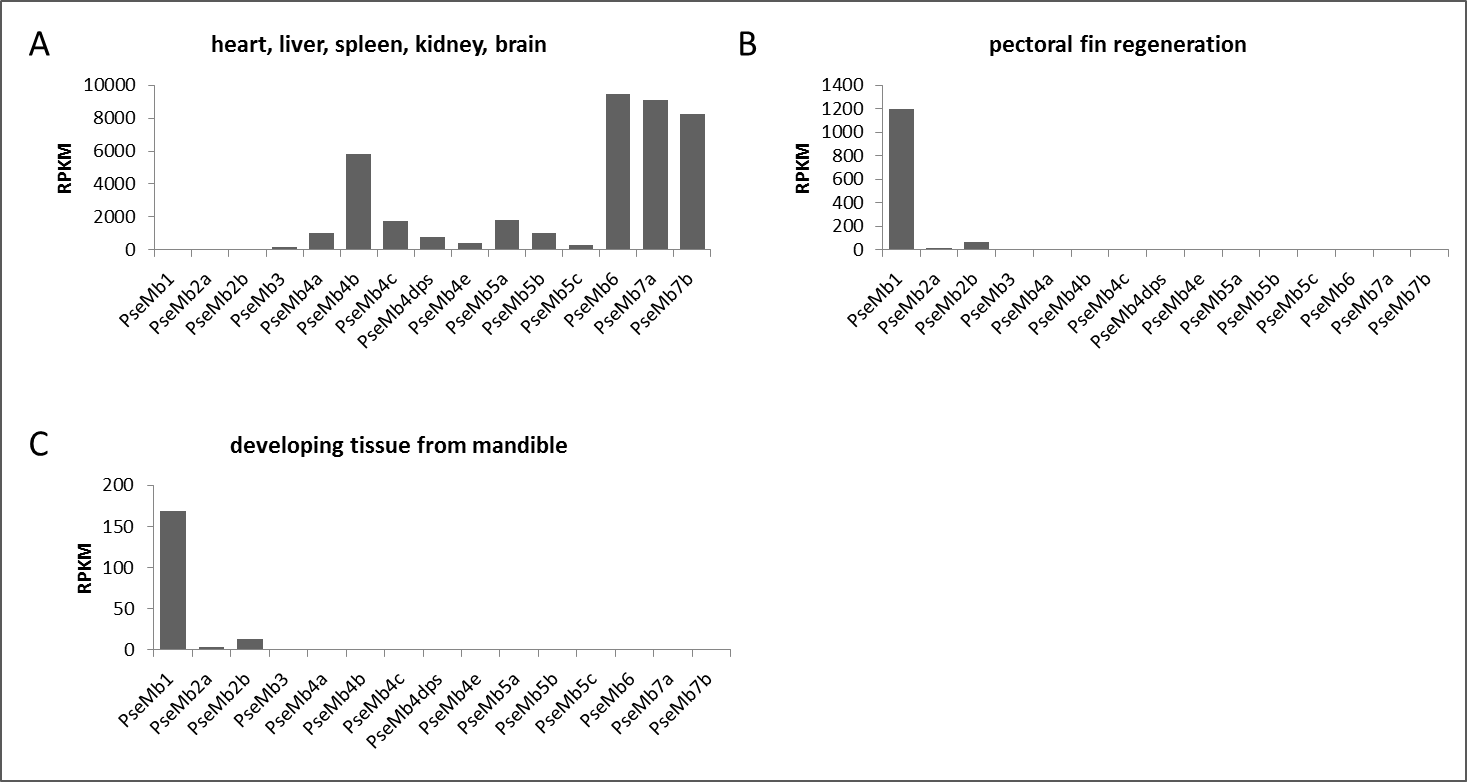


Supplementary Figure 3

Gene expression of gray bichir Mbs were estimated by RNA-Seq and are displayed as RPKM values. (A) Expression of PseMbs in a mixture of heart, liver, spleen, kidney and brain. (B) Expression of PseMbs in a developing jawbone and (B) in a regenerating pectoral fin (14 dpf).


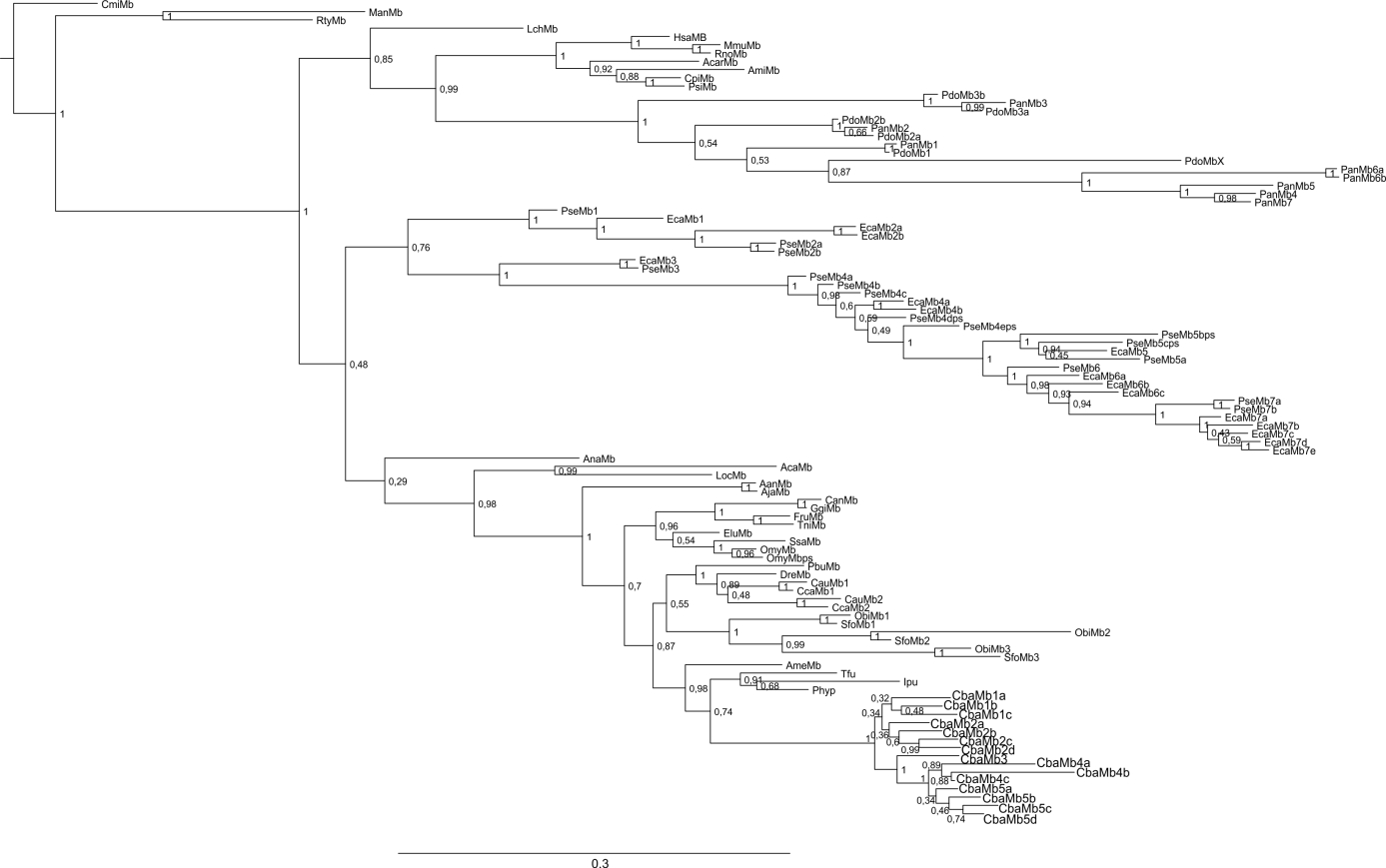


Supplementary Figure 4

Original phylogeny of multiple Mb genes in bony fish (Osteichthyes) without collapsed areas. The pedigree was calculated using Bayesian analysis using the Dayhoff + I + G model. The bar represents 0.3 substitutions per amino acid position. The single copy Mb sequence of the Australian ghost shark serves as the outer group. The numbers at the nodes are the Bayesian posterior probabilities. Nodes that are shown as collapsed (triangle) in Figure 1 include the taxa marked bold. **AanMb: Anguilla anguilla**, **AcaMb: Amia calva**, **AcarMb: Anolis carolinensis**, **AjaMb: Anguilla japonica**, AmeMb: Astyanax mexicanus, **AmiMb: Alligator mississippiensis,** AnaMb: Acipenser naccarii, **CanMb: Cryodraco antarcticus**, CauMb1: Carassius auratus, CauMb2: Carassius auratus, CbaMb*: Clarias batrachus, CcaMb*: Cyprinus carpio, CmiMb: Callorhinchus milii, **CpiMb: Chrysemys picta bellii**, DreMb: Danio rerio, EluMb: Esox Lucius, **GgiMb: Gobionotothen gibberifrons**, **HsaMB: Homo sapiens**, Ipu: Ictalurus punctatus, LchMb: Latimeria chalumnae, **LocMb: Lepisosteus oculatus**, **ManMb: Mirounga angustirostris,** **MmuMb: Mus musculus**, ObiMb*: Osteoglossum bicirrhosum, OmyMb*: Oncorhynchus mykiss, PanMb*: Protopterus annectens, PbuMb: Pantodon buchholzi, PdoMb*: Protopterus dolloi, PhypMb: Pangasianodon hypophthalmus, **PsiMb: Pelodiscus sinensis**, **RnoMb: Rattus norvegicus**, **RtyMb: Rhincodon typus**, SfoMb*:Scleropages formosus, SsaMb: Salmo salar, Tfu: Tachysurus fulvidraco, **TruMb: Takifugu rubripes**, **TniMb: Tetraodon nigroviridis**

Supplementary Table 1

Overview of the SRA data records which were screened after multiple Mb genes.

| **Spezies** | **Tissue** | **SRA-ID** | **Reads** |
| --- | --- | --- | --- |
| *Coilia nasus* (Cna) | Brain | SRR1406754 | 100 PE |
|  | Liver | SRR1406760 | 100 PE |
| Cavefish (Ame) | Brain | SRR2045427 | 100 PE |
|  | Liver | SRR2045431 | 100 PE |
|  | Heart | SRR2045429 | 100 PE |
|  | Gills | SRR2045428 | 100 PE |
|  | Testis | SRR2045435 | 100 PE |
|  | Ovary | SRR2045426 | 100 PE |
| Black ghost knifefish (Aal, Apteronotus albifrons) | Brain | SRR1532748 | 100 PE |
|  | Liver | SRR1532751 | 100 PE |
|  | Heart | SRR1532749 | 100 PE |
|  | Testis | SRR1532755 | 100 PE |
|  | Ovary | SRR1532747 | 100 PE |
| Iridescent shark (Phy, Pangasianodon hypophthalmus) | Brain | SRR1533641 | 100 PE |
|  | Liver | SRR1533645 | 100 PE |
|  | Heart | SRR1533643 | 100 PE |
|  | Gills | SRR1719651 | 114 SE |
|  | Testis | SRR1533649 | 100 PE |
|  | Ovary | SRR1533640 | 100 PE |
| Asian Seabass (Lca, Lates calcarifer) | Brain | SRR1791593 | 100 PE |
|  | Liver | SRR2179937 | 150 PE |
|  | Testis | SRR1791598 | 100 PE |
|  | Ovary | SRR1791597 | 100 PE |
| Eastern Happy (Aca, Astatotilapia calliptera) | Brain | ERR1950115 | 75 PE |
|  | Liver | ERR1953016 | 75 PE |
| Lined seahorse (Her, Hippocampus erectus) | Brain | SRR3289251 | 90 PE |
| Northern pike (Elu, Esox lucius) | Brain | SRR1228710 | 100 PE |
|  | Liver | SRR1228725 | 100 PE |
|  | Heart | SRR1228723 | 100 PE |
|  | Gills | SRR1533653 | 100 PE |
|  | Testis | SRR1533661 | 100 PE |
|  | Ovary | SRR1533651 | 100 PE |
| Ayu (Pal, Plecoglossus altivelis) | Brain | SRR1533708 | 100 PE |
|  | Liver | SRR1533712 | 100 PE |
|  | Heart | SRR1533710 | 100 PE |
|  | Gills | SRR1533709 | 100 PE |
|  | Testis | SRR1533716 | 100 PE |
|  | Ovary | SRR1533707 | 100 PE |
| Atlantic cod (Gmo, Gadus morhua) | Brain | SRR2045416 | 100 PE |
|  | Liver | SRR2045420 | 100 PE |
|  | Heart | SRR2045418 | 100 PE |
|  | Gills | SRR2045417 | 100 PE |
|  | Testis | SRR2045424 | 100 PE |
|  | Ovary | SRR2045415 | 100 PE |
| Gray bichir (Pse, Polypterus senegalus) | Mixture | SRR1612395 | 90 PE |
|  | Mandible | SRR5114773 | 51 PE |
|  | Fin 14 dpa | SRR2826833 | 76 PE |
| Reedfish (Eca, Erpetoichthys calabaricus) | Mixture | SRR3293426 | 90 PE |
|  | Gills | SRR3293424 | 90 PE |
|  | Liver | SRR3293425 | 90 PE |

Supplementary Table 2

Myoglobin sequences of Gray bichir (Polypterus senegalus).

| PseMb1 | atggctttgtcagaaggtgaatggggtttggtgctgaaggcttggggtaacgttgaatcggacccggcaggcgtcggccaagctgtcctacttcgcttgtttcacgatcataaagaaactcaaaaccatttcccgaagttcaagaacctctctgcggctgagctgcagagttctggcgatgtcaggactcatggtcaagtcgtcgttaacaagctgaccgagctgctcaagaagaaaggaaaccatgcagatatcctgaagccgctggctgagtcccactccaaaaagcacaagatcccagtccagaactttgagctgatctctgaagtcattgtgaaagtgatgtcagagaaaatgcctgactttggggcagatggtcaagccgcactcaggaaagctctgaaggttgtcgtcactgacttgggcaacttgtatgaatgttga | MALSEGEWGLVLKAWGNVESDPAGVGQAVLLRLFHDHKETQNHFPKFKNLSAAELQSSGDVRTHGQVVVNKLTELLKKKGNHADILKPLAESHSKKHKIPVQNFELISEVIVKVMSEKMPDFGADGQAALRKALKVVVTDLGNLYEC |
| --- | --- | --- |
| PseMb2a | ACTTGAGCTGAAGATGGCTATGTCAAAAAGTGACTGGGACGTGGTGCTGCAGGTGTGGCCTAAGGTTGAATCGGACCCGGCAGGCGTCGGCCAAGCTGTTCTACTTCGTTTTTTTGAAGATCATAGAGACGCTCAAGACCATTTCCCGAAGTTCAAGAACCTCTCTCGTGCTGAGCTACAGAACTGTCCCGGTGTCAGGACTCACGGTGAAGCTGTCGTCAACAGGCTGACCGAGGTGTTCAAGTTGAGAGGAGGACATGCCTCTATCGTGAAGAAGATGGCCGAGGATCACTCCAAAACGCTCAAGATCCCAGTCCAGTACCTTGAGCTGATCTGTGAAGTCATTGTGAAAGTGATGGTTGAGAAAATACCCGACTTTGGGCCAGATGGTCAAGCAGCAGTCAGGAAAGCTCTGAAGGTTTTCACCACTGACATTGGCGGCTTTTATTAATAAT | MAMSKSDWDVVLQVWPKVESDPAGVGQAVLLRFFEDHRDAQDHFPKFKNLSRAELQNCPGVRTHGEAVVNRLTEVFKLRGGHASIVKKMAEDHSKTLKIPVQYLELICEVIVKVMVEKIPDFGPDGQAAVRKALKVFTTDIGGFY |
| PseMb2b | ACTTGAGCTGAAGATGGCTATGTCAGAAGGTGACTGGGATGTGGTGCTGAAGGTGTGGGCTAAGGTTGAATCGGACCCGGCAGGCGTCGGCCAAGCTGTTCTACTTCGTTTTTTTGAAGATCATAGAGACGCTCAAGACCATTTCCCGAAGTTCAAGAACCTCTCTCGCGCTGAGCTACAGAACTGTCCCGGTGTCAGGACTCACGGTGAAGCTGTCGTCAACAGGCTGACCGAGGTGTTCAAGTTGAGAGGAGGACATGCCGCCATCGCGAAGAAGATGGCCGCGGATCACTCCAAAACGCTCAAGATCCCACACCAGTACTTTGAGCTGATCTCTGAAGTCATTATGAAAGTGATGGTTGAGAAAATACCCGACTTTGGGCCAGATGGTCAAGCAGCAGTCAGGAAAGCTCTGAAGGTTTTCACCACTGACATTGGCGGCTTTTATTAATAAT | MAMSEGDWDVVLKVWAKVESDPAGVGQAVLLRFFEDHRDAQDHFPKFKNLSRAELQNCPGVRTHGEAVVNRLTEVFKLRGGHAAIAKKMAADHSKTLKIPHQYFELISEVIMKVMVEKIPDFGPDGQAAVRKALKVFTTDIGGFY |
| PseMb3 | ATGGCTGCAGCTGATTTCGATGCGGTGCTGGCCTCTTGGGGACCCGTAGAAGCA  GATTCGCCAGGATACGGGGAAGCCGTGCTCGTTCGCCTGTTCACTGAAAATCCAGAGACCCAGAAACTGTTCCCCAAGTTCAAAAACCTCTCACAGGGCGAGCTGTCGGGCAATGCCAATATCAAGGCTCATGGAAATGTTGTCCTGTCCAAGCTGACTGCGTTGATCAAACAGAAGGGCGATCACGCTGCTCTCCTTAAGCCTCTGGCAGAAAGCCATGCTCTGCATCACAAGATTCCCAGGAAGAACTTTGAGCTTATCTCAGAGATCATTGTCAAGGTGGTTGCAGAAAAGAACTCTGCGTTCAATGCTGATGCTCAAGCTGCCCTAAGGAGAGTGCTGAAGGTGGTGGTTGCTGACCTGGGCTGCTTGTACGATGAACATGGATATAAAGAGTAA | MAAADFDAVLASWGPVEADSPGYGEAVLVRLFTENPETQKLFPKFKNLSQGELSGNANIKAHGNVVLSKLTALIKQKGDHAALLKPLAESHALHHKIPRKNFELISEIIVKVVAEKNSAFNADAQAALRRVLKVVVADLGCLYDEHGYKE |
| PseMb4a | ATGAGTGCTTCTGCTGGTGATTGTGAGACTGTGCTCTCTTTCTGGACTCCCGTGGATGCTGATCCCAGGTGCTATGGGGAGATTATTCTCCTACGCTTGTTTGAGACCCATCCAGATGTTCAGAAGCTGTTCCCCAAGTTTGTTGCTCTTTCCAAAGAGCAGCTGCAGAACAATCCTGATCTCCAGGCCCATGGGGAAATTGTGGTTTGCAAGCTGACAGAAATCCTGAAAGCTAAGGGGGAACATAAGGCAATCTTGAAGGCTCTAGCAGAAAGTCATGCCAAGCAGCACAAGATTCCTCTGGTTAACTTTCAGATCATCAGTGAAGTCATTGTCACAGTGGCAGCAGAGAAGCTTGATGGCTTTGGTTCTGATGCTAAAACAGCCCTGAAGAATGTGCTGAAGACAATTCAGATTGACTTGGGAGCTTGTTATGAAGAGCTTGGATTTAAAGAATAG | MSASAGDCETVLSFWTPVDADPRCYGEIILLRLFETHPDVQKLFPKFVALSKEQLQNNPDLQAHGEIVVCKLTEILKAKGEHKAILKALAESHAKQHKIPLVNFQIISEVIVTVAAEKLDGFGSDAKTALKNVLKTIQIDLGACYEELGFKE |
| PseMb4b | ATGAGTTCTTGTGTTGCTGATTGTAAGACTGTGCTGTCTTTCTGGGCTCCCGTGGAGGCTGATCCTAGATGCTATGGGGAGGTTATTCTTCTACGCTTGTTTGAGACCCATCCAGATGTTCAGAAGCTGTTCCCCAAGTTCGTTGGCCTTTCCAAAGAGCAGCTGCAGAACAATCCTGCTGTCCAGGCCCATGGGGAAATTGTGGTTTGCAAGCTGACAGAAATCCTAAAAGCAAATGGGGAACACAAGGAAATCTTGAAGGCTTTAGCAGAAAGCCATGCCAAGCAGCACAAGATCCCTCTGGTTAACTTTCAGATCATCAGTGAAGTCATTGTCACGGTGGCAGCAGAGAAGCTTGACCGCTTTGGTCCTGATGCTAAAACAGCCCTGAAGAATGTGCTGAAGACAATTCAGATTGACTTGGGAGCTTGTTATGAAGAGCTTGGATTTAAAGAATAG | MSSCVADCKTVLSFWAPVEADPRCYGEVILLRLFETHPDVQKLFPKFVGLSKEQLQNNPAVQAHGEIVVCKLTEILKANGEHKEILKALAESHAKQHKIPLVNFQIISEVIVTVAAEKLDRFGPDAKTALKNVLKTIQIDLGACYEELGFKE |
| PseMb4c | ATGAGTGCTTGTGTTGCGGATTGTAAGACTGTGCTCTCTTTCTGGGCTCTCGTGGATGCTGATCCCAGATGCTATGGGGAGATTATTCTCCTACGCTTGTTTGAGACCCATCCAGATGTCCAGACGCTGTTCCCCAAGTTTGTTGGCCTTTCCAACGAGCAGCTGCAGAACAATCCTGATGTCCAGGCCCATGGGGAAATTGTGGTTTGCAAGCTGACAGAAATCCTGAAAGCGAATGGGGAACACAAGGAAATCGTGAAGGCTCTGGCAGAAAGTCATGCCAAGCAGCACAAGATTCCTCTGGTTAACTTTCAGATCATCAGTGAAGTCATTGTCACGGTGGCAACAGAAAAGCTTGATGGCTTTGGTCCTGATGCTGAAACCGCCCTGAAGAATGTGCTGAAACAGATTCAGATTGACTTGGGAGCTTGCTATGATGAGCTTGGATTTAAATAA | MSACVADCKTVLSFWALVDADPRCYGEIILLRLFETHPDVQTLFPKFVGLSNEQLQNNPDVQAHGEIVVCKLTEILKANGEHKEIVKALAESHAKQHKIPLVNFQIISEVIVTVATEKLDGFGPDAETALKNVLKQIQIDLGACYDELGFK |
| PseMb4dps | ATGAGTTCTTGTGTTGCTGATTGTAAGGCTGTGCTGTCTTTCTGGGCTCCCGTGGAGGCTAATCCCAGATGCTATGGGGAGATTATTCTTCTACGCTTGTTTGAGACCCATCCAGATGTCCAGAAGCTGTTCCCCAAGTTTGCCGACCTTTCCAAAGAGCAGCTGCAGAACAATCCTTGTGTCCAGGCCCATGGGTAAATCGTGGTTTGCAAGCTGACAGAAATCCTAAAAGCGAATGGGGAACGCAAGGAAATCAGGAAGGCTCTGGCAGAAAGTCATGCCAAGCAGCACAAGATCCCTCTGGTTAACTTTCAGATCATCAGTGAAGTCATTGTCACAGTGGCAGTAGAGAAGCTTGACAGCTTTGGTCCTGATGCTAAAA | MSSCVADCKAVLSFWAPVEANPRCYGEIILLRLFETHPDVQKLFPKFADLSKEQLQNNPCVQAHGIVVCKLTEILKANGERKEIRKALAESHAKQHKIPLVNFQIISEVIVTVAVEKLDSFGPDAKX |
| PseMb4eps | ACGGGTGCTTGTACTGCTGATTGTAAGACTGTGCTCTCTTTCTGGGCTCCCGTGGAGGCTAATCCCAAATATTATGGAGAGATTATTATCCTACGCTTGTTTGAGACACATCCAGATGTCCAGAAGCTGTTCCCTAAGTTTGCTGAACTTTCCAAAGAGCAGCTGCAGAACAATCCTGGTGTCCAGGCCCATGGGGAAATTGTGGTTTGCAAGCTGACAGAAATCCTAAAAGGGGAACCTGAGGAAGTTGTGAAGGCTTTGGCTGAAACTCATGCCAAGCAGCACAAGATCCCTCTGGTTAACTTTCAGATCATCAGTGAAGTCATTGTCATGGTGGCAGCAGAGAAGCTTGATGGCTTTGGTCTTGATGCTCAAACAGCCCTGAAGAATGTGCTGAAGCAGTTTCAGATTAGAATGGGAGCTTGCTATGAGGAGCTTGGATTTAATAAGTAG | TGACTADCKTVLSFWAPVEANPKYYGEIIILRLFETHPDVQKLFPKFAELSKEQLQNNPGVQAHGEIVVCKLTEILKGEPEEVVKALAETHAKQHKIPLVNFQIISEVIVMVAAEKLD  GFGLDAQTALKNVLKQFQIRMGACYEELGFNK |
| PseMb5a | ATGTGCGATTGTACTGAGGATTTTGATAAAGTGCGCAGTTTGTGGGCTCCGATGAAAGCTAATCCTAAGTACTATGGGGAGCTAATTCTTCAGCGCTTGTTTGATACCAATCCAGATGTTCAGAAGCTGTTCCCTAAGTTTGCTGACCTTTCCAAAGAGCAGCTGCAGAACAATCCTGACCTCCAGGCCCATGGGGAAATTGTTGTCTGCAAGCTGACAGAATATGTGGAAAAAGGAGAAGAGTCACTTGTGAAGAAGCTGGGAGAAAGTCATGCCAAGCAGCACAAGATCCCTCGGGCCAACTTTCAGATAATCAGTGATGTGATTGTTCTGGTGGCAGCAGAGAAGATTGATGGCTTTGGCACTGATGTTCAAACAGCCCTGAAGAATGTGATGAAGGCGTTTCAAACTGGCATGGGAGCTTGCTATGATGAGCTAGGATTTGATCGATGA | MCDCTEDFDKVRSLWAPMKANPKYYGELILQRLFDTNPDVQKLFPKFADLSKEQLQNNPDLQAHGEIVVCKLTEYVEKGEESLVKKLGESHAKQHKIPRANFQIISDVIVLVAAEKIDGFGTDVQTALKNVMKAFQTGMGACYDELGFDR |
| PseMb5bps | ATGTCCGCTTGTGTTGAAGATTATAAAACGGTGCTCAGTTTATGGGGTCCAGTGGAGGCTAATCCTAAATACTGTGGGGAGATTATTTTTCAACGCTTGTTTGAGACCCATCCAGATGCTCAGAAGCTGTTCCCCAAGTTTGCTGGCCTTTCCAAAGAGCAGCTGGAGAACAATCCTGACTTCCAGGCCCATGGGGAAATTGCTGTCTCCACGCTGACAGAATTTGTGCAACATCAAAAGGCAGACCAGTCATTTGTGAAGGAGCTGGCACAACGTCGTGAGGTTTGGTTTCAGAAGATGAACTTTTCGATCTTTAGTGAAATCATTGTCATGGTAGCAGCAGAGAAGATTGATGGGTTTGGAACTGATGCTCAAACAGCCCTGAAGAATGTGCTGAAGGCGTTTCAGACTGCCATGGGAGCTTGATATGATGAGCTAAGATTTGATCAATGC | MSACVEDYKTVLSLWGPVEANPKYCGEIIFQRLFETHPDAQKLFPKFAGLSKEQLENNPDFQAHGEIAVSTLTEFVQHQKADQSFVKELAQRREVWFQKMNFSIFSEIIVMVAAEKIDGFGTDAQTALKNVLKAFQTAMGAYDELRFDQC |
| PseMb5cps | ATGTGCGCTTGTGCTGAGAATTTAAAGAAGGTGCTCAGTGTCTGGGGTCCCGTGAAGGCTAATCCCAAGTACTATGGGGCGATTATTCTTCAGTGATGTTCAGAAGCTGTTCCCTAAGTTTGTTTCCGAAGAGCAGCTGCAGAACAATCCTGACCTCCAAGCCCATGGGGGAAATCGTCGTCTGCAAACTGACAGAATATGTGCAACAGGAACCAGGAGACAAGTCATTTGTGAAGGGGCTGGCAACAAGTCATGCCAAGCAGCACAAGATCCCTTGAGCCAACTTTTGGATCATCAGTGAAGTGATTGTCATGGTTGCAGCAGAGATGATTGGTGGCTTTGGCACTGATGCTCAAACCGCCTTGAATGTGCTGAAGAAGTTTCAAACTGGCATGGGAGCTCGCTATGATGAGCTAGGATTTGATCAATGA | MCACAENLKKVLSVWGPVKANPKYYGAIILQXDVQKLFPKFXXSEEQLQNNPDLQAHGEIVVCKLTEYVQQEPGDKSFVKGLATSHAKQHKIPXANFWIISEVIVMVAAEMIGGFGTDAQTALNVLKKFQTGMGARYDELGFDQ |
| PseMb6 | ATGTGTGCCTGTGTTGAGGATTATAACAAGGTGCTAAATTTCTGGGCTCCCGTGGAGGCTAATCCCAAACTTTATGGAGAGATTATTCTTCAGCGCTTGTTTGAAACCAATCCAGATGTTCAGAAGCTGTTCCCCAAGTTTGCTGCCCTTTCCAAGGAGCAACTGCAGAACAATCCTGACCTCCAGACCCATGGGGAAATTGTTGTCCGCAAGCTGACAGAATTTCTGAAAAAGAAACGGGAACACCAGGAACTCGTGAGTGATCTGGCAAAAAGTCATGCCCAGCAGCACAAGATCCCTCGGGTCAATTTTCAGATCATCAGTGAAGTCATTGTCCTAGTGGCAGCAGAAAAGATTGATGGCTTTGGTCCTGATGCTCTAACAGCAATGAAGAATGTGCTGAAGGAGTTTCAAACTGACATGGGAGCTTGCTATGATAAGCTAGGATTTGATAAATGA | MCACVEDYNKVLNFWAPVEANPKLYGEIILQRLFETNPDVQKLFPKFAALSKEQLQNNPDLQTHGEIVVRKLTEFLKKKREHQELVSDLAKSHAQQHKIPRVNFQIISEVIVLVAAEKIDGFGPDALTAMKNVLKEFQTDMGACYDKLGFDK |
| PseMb7a | ATGTGTGCTAGTGATGCGGATTTTGATGCTGTGCTTAGTTTCTGGGCACCTTTGAAGGCTGAGCCCAAAATCTACGGGGAGATTGTTCTTCAGCGCATGTTTGAGACCAAACCAGATAGTCAGAAGCTGTTCCCCAAGTTTGCTGACCTTTCCAAAGAGCAACTGCAGAACAATCCTGACCTCCAGGCCCATGGAGGAATTGTCATCTGCAAGCTGACAGAATTCCTGCAAGATAAAGGGCAGGGCAAAGCTGTGACAGATCTGGCAGAAACTCATGCCAAGCAGCACAAGATCCCTCGCGTCTACTTTCAGATCATCAGTGATGTCATTGTTGAAGTGGCAGCAGAGAAGATTGAAGGATTGAGCTCTGATGCTCTAACAGCCCTGAAGAATGTGCTGAAGATGTTTCAAACTAAAATGGGAGAGTGCTATGATGTGCTGGGGTTTGATAAATGA | MCASDADFDAVLSFWAPLKAEPKIYGEIVLQRMFETKPDSQKLFPKFADLSKEQLQNNPDLQAHGGIVICKLTEFLQDKGQGKAVTDLAETHAKQHKIPRVYFQIISDVIVEVAAEKIEGLSSDALTALKNVLKMFQTKMGECYDVLGFDK |
| PseMb7b | ATGTGTGCTAGTGCTGCGGATTTTGATGCTGTGCTTAGTTTCTGGGCACCTTTGAAGGCTGATCCCAAAATCTATGGGGAGATTTTTCTTCAGCGCATGTTTGAGACCAAACCAGATAGTCAGAAGCTGTTCCCCAAGTTTGCTGCCCTTTCCAAAGAGCAACTGCAGAACAATCCTGACCTCCAGGCCCATGGAGGAATTGTCGTGTGCAAGCTGACCGAATTCTTGCAAGATAAAGGGCAGGGCAAAGCTGTGACAGATCTGGCAGAAACTCATGCCAAGCAGCACAAGATCCCTCGCATCTACTTTCAGATCATCAGTGATGTCATTGTTGAAGTGGCAGCAGAGAAGATTGAAGGATTGAGCTCTGATGCTCGAACAGCCCTGAAGAATGCACTGAAGATGTTTCAAACTAAAATGGGAGAGTGCTATGATGTGCTGGGGTTTGATAAATGA | MCASAADFDAVLSFWAPLKADPKIYGEIFLQRMFETKPDSQKLFPKFAALSKEQLQNPDLQAHGGIVVCKLTEFLQDKGQGKAVTDLAETHAKQHKIPRIYFQIISDVIVEVAAEKIEGLSSDARTALKNALKMFQTKMGECYDVLGFDK |

Supplementary Table 3

Myoglobin sequences of Reedfish (Erpetoichthys calabaricus).

| EcaMb1 | ATGGCTATGTCAGAAGGTGAATGGAATGTGGTGCTGAAGGCGTGGGCTCACGTTGAATCGGACCCGGCAGGTATCGGCCAAGCTGTCCTACTTCGCTTGTTTGCCGATCATAAAGAAACTCTAAGCAGTTTCCCGAAGTTCAAGAACCTCTCTCCGGCTGAGCTGCAGAGTTCTGGCGATGTCAGGGCTCATGGTCTAATTGTCGTTAACAAGCTGACCGAGGTGTTCAAGAAGAAAGGAGAACATGCAAATTCCCTGAAGCCGCTGGCTGAGTCTCACTCCAAAAAACACAAGATACCAATACAGTACTTTGAGATGATCAGTGATGTCATTGTGAAAGTGATGGCTGAGAAAATAGCCGACTTTGGGGCAGATGGTCAAGCCGCAGTCAGGAAAGCGCTGAAGGTTTTCACCACTGACATTGGCATCTTCTATGAATCTTGA | MAMSEGEWNVVLKAWAHVESDPAGIGQAVLLRLFADHKETLSSFPKFKNLSPAELQSSGDVRAHGLIVVNKLTEVFKKKGEHANSLKPLAESHSKKHKIPIQYFEMISDVIVKVMAEKIADFGADGQAAVRKALKVFTTDIGIFYES |
| --- | --- | --- |
| EcaMb2a | ATGGCTATGTCAGAAGGTGAATGGAATCTGGTGCTGAAGGGATGGGCTAAAGTTGAATCGGACCCGGCAGGCGTCGGCCAAGCTGTTCTACTTCGTTTTTTTGAAGATCATCCTGAAGCTCTACCCCATTTCAAGAAGTTTGAGCACCTCTCAAAGGCTGAGCTGAAGAGCTTTGCAGGTGTCAGGACTCATGGTGAAGCTGTCGTCAACTTGCTGACCAAGATGTTCAGCACGAGAGGAAAACATGCCTCTCTCCTGAAGCCGATGGCCGAGGAACACTGCAAAACGCTCAAGATCCCAGTCAAGTACTTTGAGATGATCTGTGAAGTCATTGTGAAAGTGATGGCTGAGAGTTACCCTGACTTTGGGCCAGATGGTCAAGCTGCAACCAGGAAAGCACTGAAGATTGTCTGCAAAGACATAGCCGGCTATTATGAATCATGTGAATCGACCAGATAG | MAMSEGEWNLVLKGWAKVESDPAGVGQAVLLRFFEDHPEALPHFKKFEHLSKAELKSFAGVRTHGEAVVNLLTKMFSTRGKHASLLKPMAEEHCKTLKIPVKYFEMICEVIVKVMAESYPDFGPDGQAATRKALKIVCKDIAGYYESCESTR |
| EcaMb2b | ATGGCTATGTCAGAAGGTGAATGGAATCTGGTGCTGAAGGGATGGGCTAAAGTTGAATCGGACCCGGCAGGCGTCGGCCAAGCTGTTCTACTTCGTTTTTTTAAAGATCATCCTGAAGGTAAATCCCATTTCCCGAAGTTCGAGCACCTCTCTCTCGCTGAGCTACAGACCTATGCAGGTGTCAGGACTCATGGTGAAGCTGTCGTCAACTTGCTGACCAAGATGTTCAGCACGAGAGGAAAACATGCCTCTCTCCTGAAGCCGATGGCCGAGGAACACTGCAAAACGCTCAAGATCCCAGTCAAGTACTTTGAGATGATCTGTGAAGTCATTGTGAAAGTGATGACTGAGAGTTACCCTGACTTTGGGCCAGATGGTCAAGCTGCAACCAGGAAAGCACTGAAGATTGTCTGCAAAGACATAGCCGGCTATTATGAATCATGTGAATCGACCAGATAG | MAMSEGEWNLVLKGWAKVESDPAGVGQAVLLRFFKDHPEGKSHFPKFEHLSLAELQTYAGVRTHGEAVVNLLTKMFSTRGKHASLLKPMAEEHCKTLKIPVKYFEMICEVIVKVMTESYPDFGPDGQAATRKALKIVCKDIAGYYESCESTR |
| EcaMb3 | ATGGCTGCAGCTGATTTTGATGCGGTGCTGGCCTCTTGGGGACCCGTAGAAGCAGATTCGCCAGGATACGGGGAAGCCGTGCTTGTTCGCCTGTTCACTGATCATCCAGAGTCCCAGAAACTGTTCCCCAAGTTCAAAAACCTCTCGCAGGGTGAGCTGTCAGGCAATGCAGGTATCAAGGCTCATGGAAATGTTGTCCTGTCCAAGCTGACTGCATTGATCAAACAGAAGGGTGATCACGCTGCTCTCCTTAAGCCTCTGGCAGAAAGCCATGCTCTGCAACACAAGATTCCTCGGAAGAACTTTGAGATTATCTCAGAGGTCATTGTCAAGGTGGTTGCAGAAAAGAACTCTGCTTTCAATGCTGATGCTCAAGCTGCCCTAAGGAGGGTGCTGAAGGTGGTGGTTGCTGACCTGGGCTGCTTCTACGATGAACATGGATATAAAGAGTAA | MAAADFDAVLASWGPVEADSPGYGEAVLVRLFTDHPESQKLFPKFKNLSQGELSGNAGIKAHGNVVLSKLTALIKQKGDHAALLKPLAESHALQHKIPRKNFEIISEVIVKVVAEKNSAFNADAQAALRRVLKVVVADLGCFYDEHGYKE |
| EcaMb4a | ATGAGTGCTTGTGATGCTGATTGTGCGACTGTGCTCTCTTTCTGGGCTCCTCTGGAGGCTGATCCCAGATGCCATGGGGAGATTATTCTTCTACGCTTGTTTGAGACCCATCCAGATGTTCAGGAGCTGTTCCCCAAGTTTGTTGACCTTTCCAAAGAGCAGCTGCAGAACAATCCTGGCGTCCAGGCCCATGGGGAAATTGTGGTTTGTAAGCTGACAGAAATCCTGAAAGCAAATGAGAAACGCAAGGAAATCATCAAGGCTCTAGCAGAAAGTCACGCCAAGCAGCACAAGATCCCTCTGGTTAACTTTCAGATCATCAGTGAAGTCATTGTTACAGTGGCAACAGAGAAGCTTGACGGTTTTGGTCCTGATGCTCAAACAGCCCTGAAGAATGTGCTGAAGCAGTTTCAGATTGACTTGGGAGCTTGCTATGACGAGCTTGGGTTTAAACCATAG | MSACDADCATVLSFWAPLEADPRCHGEIILLRLFETHPDVQELFPKFVDLSKEQLQNNPGVQAHGEIVVCKLTEILKANEKRKEIIKALAESHAKQHKIPLVNFQIISEVIVTVATEKLDGFGPDAQTALKNVLKQFQIDLGACYDELGFKP |
| EcaMb4b | ATGAGTGCTTGTGAGGCTGATTGTAAGACTGTGTGCTCTTTCTGGGCTCCCGTGGAGGCTGATCCCAAATGCTATGGGGAGATTGTTTTACAACGCTTGTTTGAGACCCATCCAGATGTTCAGACACTGTTCCCGAAGTTTGTTGACCTTTCCAAAGAGCAGCTGCAGAACAATCCTGGTGTCCAGGCCCATGGGGAAATTGTGGTTCGCAAGCTGACAGAAATCCTGAAAGCAAATGGGAAACGCAAGGAAATCATCAAGGATCTGGCAGAAAGTCATGCCAAGCAGCACAAGATCCCTCTGGTTAACTTTCAGATCATCAGTGAAGTCATTGTCACGGTGGTAGCAGAGAAGCTTGACGGTTTTGGTCCTGATGCTCAAACAGCCCTGAAGAATGTGCTGAAGACCTTTCAGATTGACTTGGGAGCTTGCTATGATGAGCTTGGATTTAAATAG | MSACEADCKTVCSFWAPVEADPKCYGEIVLQRLFETHPDVQTLFPKFVDLSKEQLQNNPGVQAHGEIVVRKLTEILKANGKRKEIIKDLAESHAKQHKIPLVNFQIISEVIVTVVAEKLDGFGPDAQTALKNVLKTFQIDLGACYDELGFK |
| EcaMb5 | ATGTGCGCTTGTGTTGACGATTGTAAGAAGGTGCTCAGTTGCTGGGGTCCCGTGAAGGCTAATCCCAAGCAGTATGGGGAGATTATTCTTCAACGCTTGTTTGAGACCCATCCAGGTGTTCAGAAGCTCTTCCCAAAGTTTGCTGACCTTTCCAAAGAGCAGCTGCAGAGCAATCCTGACCTCCAGGCCCATGGTGAAATTGTTGTCTGCAAGCTGACAGAATATATGCAAAAACCAGAAGATCAGTCCCTTGTGCAGGAGCTGGGAAAAAGTCACGCCGAGCAGCACAAGATCCCTCGGGCCAACTTTCAGATAATCAGTGAAGTGATTGTTATGGTGGCAGCAGAGAAGATTGACGGCTTTGGCACTGATGCTCAAACCGCCCTGAAGAATGTACTGAAGGAGTTTCAGACAGCCATGGGAGCTTGCTATGATAAGCTAGGATTTGACCCATGA | MCACVDDCKKVLSCWGPVKANPKQYGEIILQRLFETHPGVQKLFPKFADLSKEQLQSNPDLQAHGEIVVCKLTEYMQKPEDQSLVQELGKSHAEQHKIPRANFQIISEVIVMVAAEKIDGFGTDAQTALKNVLKEFQTAMGACYDKLGFDP |
| EcaMb6a | ATGTGTGCTTGTGTTGCGGATTATGAAAAGGTGCTAAATTTCTGGGCTCCCTTGGAGGCGAATCCCAGGCTTTATGGAGAGATTATTCTTCAGCGCTTGTTTGAGACCAATCCAGATGTTCAGAAGCTGTTCCCAAAGTTTGCTGCCCTTTCCAAAGAGCAACTGCAGGACAATCCTGACCTCCAGGCCCATGGGGGAATTGTTGTCTGCAAGCTGACAGAATTTCTAAAACAGAAAAAGGAACACAAGGACCTTGTGGGTGATCTGGCAGAAAGTCATGCCAAGCAGCACAAGATCCCTCGAATCAACTTTCAGATCATCGGTGAAGTCATTGTCATAGTGGCAGCAGAGAAGATTGATGGCTTTGGAACTGATGCTCAAACAGCATTGAAGAATGTGCTGAAGGAGTTTCAAACAGACATGGGAGCTTGCTATGATGCGTTCGGCTATGATCAATGA | MCACVADYEKVLNFWAPLEANPRLYGEIILQRLFETNPDVQKLFPKFAALSKEQLQDNPDLQAHGGIVVCKLTEFLKQKKEHKDLVGDLAESHAKQHKIPRINFQIIGEVIVIVAAEKIDGFGTDAQTALKNVLKEFQTDMGACYDAFGYDQ |
| EcaMb6b | ATGTGTGCTTGCGTTGAGGATTATAACAAGGTGCTCCGTTTCTGGGCTCCCCTGGAGGCTGATCCCAAACTCTATGGAGAGATTATTCTTCAACGCTTGTTTGAGACCAATCCAGATGTTCAGAAGCTGTTCCCAAAGTTTGCTGGCCTTTCCAAAGAGCAACTGCAGAACAATCCTGACCTCCAGGCCCATGGGGGAATTGTTGTCTGCAAGCTGACAGAATTTCTGCAAAAGGAAAAGCAACGCAAGCAACTTATGAATGATCTGGCAGAAAGTCATGCCAAGCAGCACAAGATCCCTCGGATCAACTTTCAGATCATCAGTGAAGTCATTGTCCTAGTGGCAGCAGAGAAGATTGAGGGCTTTGGTTCTGATGCTCAAACAGCAATGAAGAATGTGCTGAAGGAATTTCAGACTGGCATGGGAGCTTGCTATGATGTGCTAGGATTTGATCCATGA | MCACVEDYNKVLRFWAPLEADPKLYGEIILQRLFETNPDVQKLFPKFAGLSKEQLQNNPDLQAHGGIVVCKLTEFLQKEKQRKQLMNDLAESHAKQHKIPRINFQIISEVIVLVAAEKIEGFGSDAQTAMKNVLKEFQTGMGACYDVLGFDP |
| EcaMb6c | ATGTGTGCTTGTATTGAGGATTATGATAAGGTGCTAAGTTTCTGGGCTCCCCTGGAGGCAAATCCCAAGCTTTATGGAGAGATTATTCTTCAGCGCTTGTTTGAGACCAAACCAGATAGCCAGAAGCTGTTCCCCAAGTTTGCTGCACTTTCTAAAGAGCAGCTGCAGAACAATCCTGACCTCCAGGCCCATGGGGGAATTGTCATCTGCAAGCTGACAGAATTTCTGCATTCGAAACTGCAACACCAGCAACTTATGAAAGATCTGGCAGAAAGTCATGCCAAGCAGCACAAGATCCCTCGGGTCAACTTTCAGATCATTGGTGAAGTAATTGTCATAGTGGCAGCAGAAAAGATTGATGGCTTTGGTCCTGATGCTCAAACAGCATTGAAGAATGTGCTGAAGGAGTTTCAAACTGTCATGGGAGCTTGCTATAATGAGCTAGGAGTTGATCTGTGA | MCACIEDYDKVLSFWAPLEANPKLYGEIILQRLFETKPDSQKLFPKFAALSKEQLQNNPDLQAHGGIVICKLTEFLHSKLQHQQLMKDLAESHAKQHKIPRVNFQIIGEVIVIVAAEKIDGFGPDAQTALKNVLKEFQTVMGACYNELGVDL |
| EcaMb7a | ATGTGTTCTAGCACTGAGGAATATGATGCTGTGCTTAGTTTCTGGGGTCCTTTGAAGGCTGATCCCAAAAGCTATGGGGAGATCGTTCTTCAGCGCTTGTTTGAGACCAAACCAGATAGCCAGAAGCTGTTCCCCAAGTTTGCTGCCTTTTCCAAAGAGCAGCTCCAGAACAATCCTGACCTCCAGGCCCATGGGGGAATTGTCCTCTGCAAGCTGACAGAATTCCTGCAAGACAGAGGACAGGACAAGAAAATTCTGAAGGATCTGGCAGAATCTCATGCCAAGCAGCACAAGATCCCTCGGGTCTACTTTCAGATCATCAGTGATGTCATTTTTGAAGTGGTAGCAGAGAAGATTGAAGGCTTTGACACTGATGCTCAAACAGCCCTGAAGAATGTGCTGAAGACCTTTCAAACTCAAATGGGAGAATGCTATGATGAGCTGGGATTTGATTAA | MCSSTEEYDAVLSFWGPLKADPKSYGEIVLQRLFETKPDSQKLFPKFAAFSKEQLQNNPDLQAHGGIVLCKLTEFLQDRGQDKKILKDLAESHAKQHKIPRVYFQIISDVIFEVVAEKIEGFDTDAQTALKNVLKTFQTQMGECYDELGFD |
| EcaMb7b | ATGTGTGCTAGTGGTGAGGAATATGATGCTGTGCTTAGTTTCTGGGATCCTTTGAAGGCTGATCCCAAAAGCTATGGGGAGATCGTTCTTCAGCGCTTGTTTGAGACTAAACCAGATAGCCAGAAGCTGTTCCCCAAGTTTACTGCCCTTTCCAAAGAGCAGCTCCAGAACAATCCTGACCTCCAGGCCCATGGGGGAATTGTCGTCTGCAAGCTGACAGAATTCATGCAAGGTGGAGGACGGGACAAGAAAACTCTGAAAGATCTGGCTGAATCTCATGCCAAGCAGCACAAGATCCCTCGGATCTACTTTCAGATCATCAGTGATGTCATTTTTGAAGTGGTAGTAGAGAAGATTGAAGGCTTTGGCACTGATGCTCAAACAGCCCTGAAGAATGTGCTGAAGACCTTTCAAACTCAAATGGGAGAATTCTACGATGAGCTGGGATTTGATGATAAGTAA | MCASGEEYDAVLSFWDPLKADPKSYGEIVLQRLFETKPDSQKLFPKFTALSKEQLQNNPDLQAHGGIVVCKLTEFMQGGGRDKKTLKDLAESHAKQHKIPRIYFQIISDVIFEVVVEKIEGFGTDAQTALKNVLKTFQTQMGEFYDELGFDDK |
| EcaMb7c | ATGTGTGCTAGTGCTGCGGAATTTATTTCTGTGCTTAGTTTCTGGGGTCCTTTGAAGGCTGATCCCAAAAGCTATGGGGAGATTGTTCTTCAGCGCTTGTTTGAGACCAAACCAGATAGCCAGAAGCTGTTCCCCAAGTTTGCTGCCCTTCCCAAAGCGCAGCTGCAGAACAATCCTGACCTCCAGGCCCATGGGGGAATTGTCTTCTGCAAGCTGACAGAATTCCTGCAAGACGGAGGACAGGACAAGAAAATTCTGAAGGATCTGGCAGAATCTCATGCCAAGCAGCACAAGATCCCTCGGGTCTACTTTCAGATCATCAGTGATGTCATTTTTGAAGTGGTGGCCGAGAAGATTGAAGGCTTTGGCCCTGATGCTCAAACAGCCCTGAAGAATGTGCTGAAGACCTTTCAAACTCAAATGGGAGAATGCTATGATGAGCTGGGA | MCASAAEFISVLSFWGPLKADPKSYGEIVLQRLFETKPDSQKLFPKFAALPKAQLQNNPDLQAHGGIVFCKLTEFLQDGGQDKKILKDLAESHAKQHKIPRVYFQIISDVIFEVVAEKIEGFGPDAQTALKNVLKTFQTQMGECYDELG |
| EcaMb7d | ATGTGTGCTAGTGCTGAGGAGTTTATTGCTGTGCTTAGTTTCTGGGATCCTTTGAAGGCTGATCCCAAAAGCTATGGGGGGATTGTTCTTCTGCGCTTGTTTGAGACCAAACCAGATAGTCAGAAGCTGTTCCCCAAGTTTGCTGACCTTTCCAAAGAGCAGCTGCAAAACAATCCTGACCTCCAGGCCCATGGAGGAATTGTCCTCTGCAAACTGACGGAATTCCTGCAAGACGGAGGACAGGACAAGAAAATTCTGAAGGATCTGGCAGAATCTCATGCCAAGCTGCACAAGATCCCTCGAATCTACTTTCAGATCATCAGTGATGTCATTTGTGAAGTGGTAGCAGAGAAGATTGAAGGCTTTGACACTGATGCTCAAACAGCCCTGAAGAATGTGCTGAAGACCTTTCAGACTCAAATGGGAGAACACTATGATGAGCTGGGATTTGATTAA | MCASAEEFIAVLSFWDPLKADPKSYGGIVLLRLFETKPDSQKLFPKFADLSKEQLQNNPDLQAHGGIVLCKLTEFLQDGGQDKKILKDLAESHAKLHKIPRIYFQIISDVICEVVAEKIEGFDTDAQTALKNVLKTFQTQMGEHYDELGFD |
| EcaMb7e | ATGTGTGCTAGTGCTGAGGAGTTTACTGCTGTGCTTAGTTTCTGGGATCCTTTGAAGGCTGATCCCAAAAGCTATGGGGAGGTTGTTCTTCAGCGCTTGTTTGAGACCAAACCAGATAGCCAGAAGCTGTTCACCAAGTTTGCTGACCTTCCCAAAGAGCAGCTACAGAACAATCCTGACCTCCAGGCCCATGGGGGAATTGTCCTCTGCAAGCTGACAGAATTCCTGCAAGGTGGAGGACGGGACAAGAAAACTCTCAAGGATCTGGCAGAATCTCATGCCAAGCAGCACAAGATCCCTCGGGTCTACTTTCAGATCATCAGTGATGTCATTTGTGAAGTGGTAGCAGAGAAGATTGAAGGCTTTGACACTGATGCTCAAACAGCCCTGAAGAATGTGCTGAAGACCTTTCAGACTCAAATGGGAGAACACTATGATGAGCTGGGATTTGATTAA | MCASAEEFTAVLSFWDPLKADPKSYGEVVLQRLFETKPDSQKLFTKFADLPKEQLQNNPDLQAHGGIVLCKLTEFLQGGGRDKKTLKDLAESHAKQHKIPRVYFQIISDVICEVVAEKIEGFDTDAQTALKNVLKTFQTQMGEHYDELGFD |

Supplementary Table 4

Myoglobin amino acid sequences of fish that were used for phylogenetic analyses.

| >AanMb | European eel  (*Anguilla anguilla*) | MTDFELVLKAWKPIEADLKGNGGVVLTRLFQEHPETQQLFPKFAAIAPGDLAGNAAISEHGCTVLTKLGDLLHAKGNHADILKPLAKTHATQHKIKLQNFQLITEVIVKLMGEKGVDAAGQEAVRKVMLAVIGDIDNFYKVLGF |
| --- | --- | --- |
| >AcaMb | Bowfin  ***(****Amia calva*) | MSLSSGEWDLVLKAFGCVKADAAGKGGEVLRGLFKAHPATIQLFPKFRSLSEAEIQESAAVPNHGATVINKLGDLLSRRGEYGPELKPMAQSHAHTHKIPLENFTLISEVIVQLMKGRXXXXXXXXXXXXXXXXXXXXXXXXXXXXXXXXXXX |
| >AcarMb | American chameleon  (*Anolis carolinensis*) | MELSDQEWQKVIDIWGKVEPEIPAYGQVVILRLFEQHPETQEKFDKFKNLKSLDEMKNSEDLKKHGTIVLTALGKILKQKRQHEAELAPLAQSHATKHKIPVKYLEFISEVIVGVIAEKRSADFGAESQAAMRKALELFRNDMDRKYKELGFQGE |
| >AjaMb | Japanese eel  (*Anguilla japonica*) | MTDFELVLKAWKPIEADLKGNGGVVLTRLFQEHPETQTLFPKFAAIAPGDLAGNAAISEHGCIVLTKLGDLLHAKGNHADILKPLAKTHATQHKIKLHNFQLITEVIVKLMGEKGVDAAGQEAVRKVMQAVIGDIDNFYKEFGF |
| >AmeMb | Cave fish  (*Astyanax mexicanus*) | MADFDAVLKVWGAVEADFTGYGGEVLSRLFLEYPETQKLFPKFVIPRGEVVGNAAIAAHGVTVLKKLGELLKAKGNHASILKPLATTHANQHKIGLNNFKLITEILAKVLGEKAGLDGAGQDSLRKVLGIVINDIDGYYKELGFAG |
| >AmiMb | American alligator  (*Alligator mississippiensis*) | MQWKHVLDIWTKVESKLPEHGHEVIISVLQVHPETQERFEKFKHMKTADEMKSSEKMKEHGTNVFTALGNILKQKGNHAEVLKPLAKSHAHKIPVKYLEFISEIIVKVIAEKYPADFGADSQAAMRKALELFRNDMASKYFGYG |
| >AnaMb | Adriatic sturgeon  (*Acipenser naccarii*) | RVNGTLVLRAWGPVESDLAGHGQVVLLRLFKDHPETLQLFPKFKSLTAGELAGSADIKAHGNTVISAVGDLLKQKGGHATLLRPLGESHAKKHKIPLANFKLICDVIVTVMKEKYSDFGPDSQAAMSKALDLIFAGMGPLYQEFGFAG |
| >CanMb | Long-fingered icefish  (*Cryodraco antarcticus*) | MADFDMVLKCWGPVEADHATHGSLVLTRLFTEHPETLKLFPKFAGIAHGDLAGDAGVSAHGATVLKKLGDLLKARGGHAALLKPLSSSHATKHKIPIINFTLIAEVIGKVMEEKAGLDAAGQTALRNVMAIIITDMEADYKELGFTE |
| >CauMb1 | Goldfish  (*Carassius auratus*) | MADHELVLKCWGVVEADFEGTGGEVLTRLFKQHPETQKLFPKFVGIAQSDLAGNAAVNAHGATVLKKLGELLKARGDHAAILKPLATTHANKHKIALNNFRLITEVLVKVMAEKAGLDAAGQTALRKVMEAVIGDIDTYYKEFGFAG |
| >CauMb2 | Goldfish  (*Carassius auratus*) | MADYERFLKCWGAVEADYTGNGGEVLTRLFKAHPDTQKLFPKFKGISQSELAGNALVAAHGATVLKKLGELLRAKGDHAAILHPMATTHANKHKITLNNFRLITEVLVEVMKEKAGLDSAGQGALKRIMDCIIHDIDRYYKEIGFAG |
| >CbaMb1a (g8413.t1) | Walking catfish  (*Clarias batrachus*) | GSQAGNAAIADHGKTVLTKLGEILKAKGSNEIIRPLATTLANKHKIVLNNFKLITEVIINVFGEKGVWDAATQDAFRKVMGVVVNEIDCVYKELGFTG |
| >CbaMb1b (g2544.t1) | Walking catfish  (*Clarias batrachus*) | MSDCELILASWGKVENNLADYGGEVLTRLFTEHPDTQKLFPMFVGIPSGELAGNEGVADHGKTVLTKLGEILKAKCSSDVIKPLATTHANKHKIALNNFKLITEVIIKVFGEKSVWDAATQEAFRKVMGGVVTEIDCVYKEVGFTG |
| >CbaMb1c (g8412.t1) | Walking catfish  (*Clarias batrachus*) | MCNCDLVLATWGKVEGNLADFGGEVLTRLFTEHPDTKKLFPKFVGIPCGELAGNAAIADHGKTVLTKLGEILKAKGSDVLKPLATTHANKHKIALNNFKLITEVIIKVFGEKGVWDAATQDAFRQVMSGVVNEIDCIYKELGFTG |
| >CbaMb2a (g2543.t1) | Walking catfish  (*Clarias batrachus*) | LFTEHPDTQELFPKFVGIPSGELAGNEGVADHGKTVLTKLGEILKAKGSNEVIKPLATTHANKHKITLNNFKLITEVIIKVFGEKGVWDTATQDAFRSVMGVVVNEIDCVYKELGFAG |
| >CbaMb2b (g7580.t1) | Walking catfish  (*Clarias batrachus*) | MSDCELILTSWGKVEGNLAGYGGEVLTRLFTEHPDTQKLFPKFVEIPCGELAGNTAVADHGKTVLTKLGEILKAKCSSDVIKPLATTHANKHKIALNNFKLITEVIIKVFGEKGVWDVAAQDAFRSVMGIVVNEIDCVYKGLGFSG |
| >CbaMb2c (g8410.t1) | Walking catfish  (*Clarias batrachus*) | MSDCKLILASWGKVESNLDGYGGEVLTRLFTEHPDTQKLFPKFAEIPHGELAGNAAIADHGKTVLMKLGEILKANGSSDVIKPLATTHANKHKIALNNFKLITEIIINVFEEKGLWDAATEDAFRSVMGVVVNEIDCVYKELGFNG |
| >CbaMb2d (>g8411.t1) | Walking catfish  (*Clarias batrachus*) | MSDCELILASWGKVESNLTAYGGEVLTRLFTEHPDTQKLFPKFVTISSSDLAGNAAIADHGKTVLMKLGEILKAQGSSGVIKPLATTHANKHKIALNNFKLITEVIVKVFGEKGLWDAATQDAFRSVMGVVVNEIDCVYKELGFNS |
| >CbaMb3 (g8414.t1) | Walking catfish  (*Clarias batrachus*) | MSDCDLILASWGKVEGNISGLGGEVLTRLFTEHPDTQKLFPKFVGIARGDLAGNAAVADHGKTVLTKLGEILKAKGSSDTIKPLATTHANKHKIGLNNFNLITEVIIQLFGEKGIWDAAAQDAFRKVMGSVVNEIGCVYKQLGFAG |
| >CbaMb4a (g21111.t1) | Walking catfish  (*Clarias batrachus*) | MSDCELILDSWGKVESNLTGYGGEVLASLFTEHPDTQKLFPKFVGIPPAELAGNAAIGEHGKTVLTKLGEILKAKGSSDVIKPLATTHANTHKISLNNFKRFFSLYFQKEQFDSCLYIVY |
| >CbaMb4b (g21110.t1) | Walking catfish  (*Clarias batrachus*) | MERIQPQTYYWRLIIYTHSLDSLFTEHPDTQKLFPKFVGISHADLAENAAVGEHGKTVLTKLGEILRAKGSGDVIKPLATTHANTHKISLNNFKLITEVIIKLFGEKXVWDAATQDAFRKVMGDVXNEIDCVYKQXGFAG |
| >CbaMb4c (g21113.t1) | Walking catfish  (*Clarias batrachus*) | MSDCELILDSWGKVESNLTGYGGEVLASLFTEHPDTQKLFPKFVGIPHAELAENAAVGEHGKTVLTKLGEILKAKGSSDVIKPLATTHANXHKIGLNNFKLITEVIIKLFGEKGVWDAATQDAFRKVMGDVVXEIDCVYKQLGFAG |
| >CbaMb5a (g21112.t1) | Walking catfish  (*Clarias batrachus*) | MSDCELILASWGKVESNLTGYGGEVLACLFTEHPDTQXLFPKFVGISLAELDGNAAIAEHGKTVLTKLGEILKAKGSSDVIKPLATTHANTHKIGLNNFKLITDVIIELFGEKGVWDAATQDAFRKVMGDVVNEIDXVYKQLGFAG |
| >CbaMb5b (g21774.t1) | Walking catfish  (*Clarias batrachus*) | QSIEEHGKTVLTKLGEILKAKGSSDVIKPLATTHANTHKIGLNNFKLITEVIIKLFGEKXVWDXAXQDXFRXXMGDVVNXIDCVYKQLGFDG |
| >CbaMb5c (g21109.t1) | Walking catfish  (*Clarias batrachus*) | MSDCELILASWEKVESNIADYGGEVLACLFTEHPDTQKLFPKFVGIPPADLAGNAAIAEHGKTVLTKLGEILQAKESSDVIKPLATTHANMHKIGLNNFKLITEVIIKLFGXXGVWDAAIQDAFRRVMGDVVNEIDCVYKQLGFTG |
| >CbaMb5d (g23112.t1) | Walking catfish  (*Clarias batrachus*) | MSDCELILASWEKVESNLADYGGEVLTCLFTEHPDTQKLFXKFVGISHADLAENAAIVEHGKTVLTKLGEILKAKGSSDVIKPLATTHANTHKIGLNNFKLITEVIIKLFXEKGVWDXATQDXFRKVMGDVVNEIDGVYKQLGFAG |
| >CcaMb1 | Common carp  (*Cyprinus carpio*) | MADHELVLKCWGGVEADFEGTGGEVLTRLFKQHPETQKLFPKFVGIAQSDLAGNAAVKAHGATVLKKLGELLKARGDHAAILKPLATTHANTHKIALNNFRLITEVLVKVMAEKAGLDAGGQSALRRVMDVVIGDIDTYYKEIGFAG |
| >CcaMb2 | Common carp  (*Cyprinus carpio*) | MADYERFLKCWGAIEADYAGHGGEVLTRLFKEHPDTLKLFPKFKGIPQSELAGDTLVASHGATVLKKLGELLRAKGDHAAILQPLATTHANKHKIALNNFRLITEVLVKVMAEKAGLDTAGQGALKRVMDCIIRDIDRYYKEIGFAG |
| >CmiMb | Australian ghost shark  (*Callorhinchus milii*) | MCDWDLINKVWAKVEEDLAGNGQTVLLRLFEEHPETKAHFPKFKDIPLGQLTSNADVKTHGNTVFKALGDVVKQKGKHASNLQALATTHINKHKIPPQNFTLITNVILKVFAEKFPGEMTAPAQEAFSKAFKAICSELEDLYKKGGFQS |
| >CpiMb | Painted turtle  (*Chrysemys picta bellii*) | MGLSDDEWHHVLGIWAKVEPDLSAHGQEVIIRLFQVHPETQERFAKFKNLKTIDELKSSEEVKKHGTTVLTALGRILKLKNNHEPELKPLAESHATKHKIPVKYLEFICEIIVKVIAEKHPSDFGADSQAAMRKALELFRNDMASKYKEFGFQG |
| >DreMb | Zebrafish  (*Danio rerio*) | MADHDLVLKCWGAVEADYAANGGEVLNRLFKEYPDTLKLFPKFSGISQGDLAGSPAVAAHGATVLKKLGELLKAKGDHAALLKPLANTHANIHKVALNNFRLITEVLVKVMAEKAGLDAAGQGALRRVMDAVIGDIDGYYKEIGFAG |
| >EluMb | Northern pike  (*Esox lucius*) | MADFEMVLSCWEPIEADYNKNGGLVLTRLFAEYPETQKLFPKFAGIAKGDLAGNAAVAAHGATVLKKLGELLKAKGNHGAILKPLATTHANKHKIPLNNFKLITEVICKVMGEKAGLDAGGQEALRRVMSVVIADIDGTYKELGFAG |
| >TruMb | Torafugu  (*Takifugu rubripes*) | MADFETVLKFWGPVEADYGAHGGIVLTRLFTENPETQKLFPKFAGITQSDLAGNAAVSAHGATVLKKLGELLKAKGNHAALLQPLANTHATKHKIPINNFKLIAEVIGKVMEEKAGLDAAGQQALKNVMATIIADIDVTYKDLGFS |
| >GgiMb | Humped rockcod  (*Gobionotothen gibberifrons*) | MADFDMVLKCWGPVEADYTTHGSLVLTRLFTEHPETLKLFPKFAGIAHGDLAGDAGVSAHGATVLNKLGDLLKARGAHAALLKPLSSSHATKHKIPIINFKLIAEVIGKVMEEKAGLDAAGQTALRNVMAVIIADMEADYKELGFTE |
| >HsaMB | Human  (*Homo sapiens*) | MGLSDGEWQLVLNVWGKVEADIPGHGQEVLIRLFKGHPETLEKFDKFKHLKSEDEMKASEDLKKHGATVLTALGGILKKKGHHEAEIKPLAQSHATKHKIPVKYLEFISECIIQVLQSKHPGDFGADAQGAMNKALELFRKDMASNYKELGFQG |
| >Ipu | Channel catfish  (*Ictalurus punctatus*) | MSDFDTVLTSWGSMEANYAAIGGEVLGRLFVEHPETQKLFPKFAGISAADAAGNPAVKAHGETVLKKLGELIKAKGNHADILKPLATSHANIHKITITNFKLISEIIIKVMAEKGLLNSGGQDAMRRVLAAVINDIDVYYKELGFAG |
| >LchMb | Coelacanth  (*Latimeria chalumnae*) | MALSEAEWGLILKVWGKAEPEAASNGKSVLLRMFQEHPDTQQHFPKFKHMTYQELQSSEELKTHGDTVLSKLGCLLKLKGNHAGDLHPLAQTHATKHKIPLHNFEIISEIIVKILAEKYPGDFGADGQAALKKALSMIIQDMGGMYKEFGFKG |
| >LocMb | Spotted gar  (*Lepisosteus oculatus*) | MVEWDMVLKAWAPVEADAAGHGAEVLTRLFKAHSGTQLLFPKFRSLTAGELVGNEELAAHGSIVVKKLGEMLKRRGKHADVLRPLAQTHVHTHKIPPDNFKHISGIFVQLMKEKXXXXXXXXXXXXXXXXXXXXXXXXXXXXXXXXXXX |
| >ManMb | Northern elephant seal  (*Mirounga angustirostris*) | MVDWEKVNSVWSAVESDLTAIGQNILLRLFEQYPESQNHFPKFKNKSLGELKDTADIKAQADTVLSALGNIVKKKGSHSQPVKALAATHITTHKIPPHYFTKITTIAVDVLSEMYPSEMNAQVQAAFSGAFKIICSDIEKEYKAANFQG |
| >MmuMb | Mouse  (*Mus musculus*) | MGLSDGEWQLVLNVWGKVEADLAGHGQEVLIGLFKTHPETLDKFDKFKNLKSEEDMKGSEDLKKHGCTVLTALGTILKKKGQHAAEIQPLAQSHATKHKIPVKYLEFISEIIIEVLKKRHSGDFGADAQGAMSKALELFRNDIAAKYKELGFQG |
| >ObiMb1 | Arowana  (*Osteoglossum bicirrhosum*) | MSDFDLVLKFWDAVEADYTAIGGDVLARLFKDHPETQKLFPKCANIPPSEVASNATVAAHGAVVLRKLGELLKARGDHASILKPLATTHANIHKISLNNFTLFTEVIVKVFAEKAGLGADGQVALRNLMAAVVTDIGGFYKELGFQA |
| >ObiMb2 | Arowana  (*Osteoglossum bicirrhosum*) | MADFDKVLKCWPAIEADPETVGGEVLNWVFIQYPETRKEFPKFDDIPTTALASHPAVKKHGGVMVRKLGEIPKAKGDHKPIVKHLATTHATIHKISLRNYKMFKEALVKVFGEKGLLDAEGQAGLGNVMDMIISETDDFYKELGVQE |
| >ObiMb3 | Arowana  (*Osteoglossum bicirrhosum*) | MSDYEKILKNWDAVEADPNGIGGEVLYTLFKDYPDTLKYFPKFAGIPPSDLATNALVAQHGGVVVKKLTELLKARGNNASILKPFATSHAKTHKIPTSNFKLITEVIVKIGADKGVLDAAGQNAFRNVMSSIIADLDTYYKELGFQG |
| >OmyMbps | Rainbow trout  (*Oncorhynchus mykiss*) | MANFDMWGPVEADYNKHGGLVLSRLFAEHPVVAGDLSGNAAVAAHGATVLMKLGELLKAKGDHAAILKPLATTHATKHRIALNNFMLITEIICNVMGEKAGLDRAGHEALRQVMGVIIADIDVTYKELGFAG |
| >OmyMb | Rainbow trout  (*Oncorhynchus mykiss*) | MADFDMVLKCWGPVEADYNKHGGLVLSRLFAEHPDTLKLFPKFAGIAAGDLSGNAAVAAHGATVLKKLGELLKAKGDHAAILKLLATTHATKHKIALNNFMLITEIICNVMGEEAGLDGAGQEALRQVMGVIIAEIDVTYKELGFTG |
| >PanMb1 | West African lungfish  (*Protopterus annectens*) | MASLSDAQWKKLQEFWVKNVEPNLTKHGQEVLVRMFVNHKSTLEYFPKFRHLTTEAEMRSNEDIRKHGNTVFTALGKLVKLKGNVEGDLRSMADSHANKHKIHLENFDIISKVIDNYFHESFPGDYGADVQDYMKATLALIVQTLTKLYKELGK |
| >PanMb2 | West African lungfish  (*Protopterus annectens*) | MTLSEAQWNNVLAFWAKHIENDPTKHGHEVLIRLFLESKAAQNLFDKFRHLGSEAEMRSCADLQKHGNTVFTALGKTLKLKGHHDADLRPMAESHSHKHKIPVENFTLICSIIDKYLHESFSDYTGDTRESLKSALGGVCHSLEKLYKEV |
| >PanMb3 | West African lungfish  (*Protopterus annectens*) | MASAAQWDTTLKFWEAHVAGDLKKHGHEALVRLFLKNKDSQKHFPKFKDLASEAEMRGSDGLKNHGETVFTALGKALQQRDGIANELRPLAVTHSQNHKIPLEEFENICEVIDVYLAEICPDYAGETRTSVKAVLDVFSQSMTTLYGEV |
| >PanMb4 | West African lungfish  (*Protopterus annectens*) | MAGLSEVQWNELLAFWDKYVAPSSSEHGKHILIRMFQTEKATQTLFSKFKDIPTSDLAVNADVKKHGGVVVDFLGKLLKLKGQNDSQLHTMAESHKNKHKIPLDYFQVISSVIDVYVNENLPEEYAPVRQSMKSALNQIANGLKDNYAKV |
| >PanMb5 | West African lungfish  (*Protopterus annectens*) | MAGLSDAQWNDLLAFFDKFIAPNSAEHGKHILIRMFDSDRATQSLFPKFKDAPAADLPKNADVKKHGGVVVDFLGKLLKQKGHNESMLHTMAETHKNKHKVLPDYFQLISSVIDVYVHENLPAEYAPVRDAMNAALKQIANTLKSNYAKV |
| >PanMb6a | West African lungfish  (*Protopterus annectens*) | MACPAKFWEENVVPDAAEHGKNILIRLYKEDPAALGFFPKYKDIPVSELGNNADVKEQGAVVVKALGELLKLKGQHESQLHAMAESHKNTYKIPVEYFPKIFKITDAYLQEKVGAAYAAIQAAMNVAFDQIADGLKTQYQTV |
| >PanMb6b | West African lungfish  (*Protopterus annectens*) | MACPAKFWEENVVPDAAEHGKNILIRLYKEDPAAQGFFSKYKDTPVSELGNNADVKEQGAVVVKALGELLKLKGQHESQLHAMAESHKNTYKIPVEYFPKIFKITDAYLHEKVGAVYAAIQAAMNVAFDQIADGLKTQYQTV |
| >PanMb7 | West African lungfish  (*Protopterus annectens*) | MAGLSEAQWNELLAFWDKYVAPSSSEHGKHILIRMFDADKATQALFSKYKDIPTSDLAANADVKKHGGVVVDFLGKLLKLKGQNDSQLHTMAESHKNKHRIPLDYFQLISTVIDVYVYENLPGEYGPVRESLKAALSQIANGLKANYAKV |
| >PbuMb | African Butterfly Fish  (*Pantodon buchholzi*) | MADHDLVLKCWGVIETDYAGYGTEVLIRLFKEHPETQKLLPKFASVPQSELAGNPAVAAHGAMVLKKLGELLKAKGNHSAIIRPLATSHANIHKIPLNNFRLISEVLVKVLAEKAILDGAGQDALRRVMGVVIGDIDIVYKEIGFNG |
| >PdoMb1 | Slender lungfish  (*Protopterus dolloi*) | MASLSDAQWKKLQEFWVKNVEPNLTKHGQEVLVRMFVNHKSTLEYFPKFRHLTTEAEMRSSEDIRKHGNTVFTALGKLLKLKGNVEGDLRSMADSHANKHKIHLENFDIISKVIDNYFHESFPGDYGADVQGYMKATLALIVQTLTKLYKELGK |
| >PdoMb2a | Slender lungfish  (*Protopterus dolloi*) | MTLSEAQWNNVLAFWAKHIDSDPTKHGHEVLIRLFLESKAAQNLFDKFRHLSSEAEMRSCADLQKHGNTVFTALGKILKLKGHHDTDLRSMAESHSKKHKIPVENFRLISNIIDKYMHDSFSDYTGDTRESFKSALGGVCHSLEKLYKEV |
| >PdoMb2b | Slender lungfish  (*Protopterus dolloi*) | MTLSEAQWNNVLAFWAKHIESDPTKHGHEVLIRLFLESKAAQNLFDKFRHLGSEAEMRSCADLQKHGNTVFTALGKTLKLKGHHDADLRPMAESHSKKHKIPVENFTLISNIIDNYLHDSFPDYTGDTRASFKSALGGVCHSLEKLYKEV |
| >PdoMb3a | Slender lungfish  (*Protopterus dolloi*) | MASAAQWDTTLNFWETHVAGDLKKHGHEALIRLFLKNKGSQQHFPIFKDLASEAEMRGCDGLQNHGETVFTKLGKALQQRDDIANELRPLAVTHSQNHKIPLEEFANICEVIDVYLAEICPDYAGETRTSVKAVLDVFVQTMKKLYGEV |
| >PdoMb3b | Slender lungfish  (*Protopterus dolloi*) | MVSAAQWDTALNFWETNVAGDLKKHGHEVLVGLFLKNKGSQQHFPKFKNLASEAEMRSSDGLQKHGETVFTTLGKALRQRDGIADELRPLAVTHSQNHKIPLEEFANICEVIDAYLAEICPDYAGETRASVKAVLDVFVQTMKKLYGEV |
| >PdoMbX | Slender lungfish  (*Protopterus dolloi*) | MVVLSEPQYNVLLSFLAEKIEPNTEKHGLEILIGLFIKSKSTQQLFPKFRNLATEAEMRSCPELKKHGDLVVKTLLNVIKKKGNHESELRKLACSHVNEHKIAVVEFKGIFGVIKDYLHTVPACTADISNSMNTVLDDIYDRLTELYKTEKK |
| >PhypMb | Striped catfish  (*Pangasianodon hypophthalmus*) | MSDFAVVLNSWGKVESDYNGYGGEVLTRLFLEQPETQKLFPKFVGIPRGELAGNAAVAAHGVTVLKKLGELIKAEGKHADILKPLATSHANIHKIALNNFKLISEIIVKVMAEKAQLDGPGQDALRRVLAVVVNDIDRYYKELGFAG |
| >PsiMb | Chinese softshell turtle  (*Pelodiscus sinensis*) | MGLSDNEWQHVLGIWAKVESDIPAHGQEVMIRLFQVHPETQSLFAKFKNLKTADEMKSSDELKKHGITVLTALGRILKQKNNHEQELKPLAESHATKHKIPVKYLEFICEIIVKVIAEKHPADFGADSQAEMRKALELFRNSMASKYKEFGFQG |
| >RnoMb | Rat  (*Rattus norvegicus*) | MGLSDGEWQMVLNIWGKVEGDLAGHGQEVLISLFKAHPETLEKFDKFKNLKSEEEMKSSEDLKKHGCTVLTALGTILKKKGQHAAEIQPLAQSHATKHKIPVKYLEFISEVIIQVLKKRYSGDFGADAQGAMSKALELFRNDIAAKYKELGFQG |
| >RtyMb | Whale shark  (*Rhincodon typus*) | MSDWENVNKVWPVVESNITAVGQKILLRLFEDHPDTKAVFPKFKEIPVEQLKNNEDLRKHGTIVLRALGNIFKQKGNHSVNVKELAETHIHKHKVPPQNFTFITNVALIILTEMYPSEMTKPMQDSFSKVFKIICSDLEQLYKAANFQG |
| >SfoMb1 | Asian arowana  (*Scleropages formosus*) | MSDFDLVLKFWDAIEADYTAIGGEVLTRLFKDHPDTQKLFPKCANIPPSEVAGNVTVAAHGAIVLRKLGELLKARGDHASILKPLATTHANIHKISLNNFTLLTEVIVKVFAEKAGLGADGQVALRNLMGVVVADIGGFYKELGFQA |
| >SfoMb2 | Asian arowana  (*Scleropages formosus*) | MADFDKVLKCWAVVEADPDAIGGEVLNCLFMEYPDTQKQFPKFAAIPPAELAGNAAVRKHGGVVVRKLGELLKAKGDHTLILKPLATTHANIHKISLNNFKMFKEALVKVFAAKGLLDADGQAALRNVMDVIIADIDGFYKELGFQG |
| >SfoMb3 | Asian arowana  (*Scleropages formosus*) | MSDYDKILKNWDAVEADPNGIGGEVLYGLFKEYPDTLKYFPKFAGIPPGDLATNPAVAQHGEIVLRKLTEILKARGNHAAILKPFANSHAKTHKIPTINFKLITDVIVKITGDKGVLDAAGQNAFRNVMSSIIADLDAFYKDANFQG |
| >SsaMb | Atlantic salmon  (*Salmo salar*) | MANYDMVLQCWEPVEADYNNHGGLVLSRLFAEHPETLTLFPKFAGIAAGDLSGNAAVAAHGATVLRKLGELLNARGDHAATLKSLATTHANKHKIPLKNFTLITNIICKVMGEKAGLDEAGQEALRQVMGVIIADINVTYMELGFAG |
| >Tfu | Yellow catfish  (*Tachysurus fulvidraco*) | MSDFDLVLKCWGKVESDYPGYGGEILTRLFLEHPESQKLFPKFVGLPQSSLAGNKDVAAHGTTVLKKLAELVKAKGQHADILKPLAASHANIHKIPLNNFKLISEIIVKVFEEKAGLDAAGQDALRRVLATVINDMDCYYKECGFAG |
| >TniMb | Spotted green pufferfish  (*Tetraodon nigroviridis*) | MGDFDMVLKFWGPVEADYSAHGGMVLTRLFTENPETQQLFPKFVGIAQSELAGNAAVSAHGATVLKKLGELLKAKGNHAAILQPLANSHATKHKIPIKNFKLIAEVIGKVMAEKAGLDTAGQQALRNIMATIIADIDATYKELGFS |

Supplementary Table 5:

Estimates of evolutionary divergence in the Mbs of lungfish (PanMbs and PdoMbs), reedfish (EcaMbs) and gray bichir (PseMbs). The number of amino acid substitutions per site from averaging over all sequence pairs within **A** and between **B** each group are shown. Standard error (SE) estimates are shown in the second column and were obtained by a bootstrap procedure (500 replicates).

| **A (within groups**) | **Average substitutions per site** | **Std. Err** |
| --- | --- | --- |
| Lungfish (PanMbs+PdoMbs) | 1.120 | 0.094 |
| Reedfish (EcaMbs) | 0.927 | 0.089 |
| Gray bichir (PseMbs) | 0.748 | 0.072 |
| **B (between groups)** |  |  |
| Lungfish – Grey bichir | 1.968 | 0.188 |
| Reedfish - Lungfish | 1.941 | 0.186 |
| Grey bichir - Reedfish | 0.834 | 0.080 |

Supplementary Table 6:

Estimates of evolutionary divergence between selected pairs of Mbs. The number of amino acid substitutions per site from between sequences are shown. Standard error estimates are shown in the last column. The rate variation among sites was modeled with a gamma distribution (shape parameter = 2,48). This analysis involved 45 amino acid sequences. All ambiguous positions were removed for each sequence pair (pairwise deletion option). There were a total of 157 positions in the final dataset.

| Reedfish | Grey bichir | Distance | Std. Err |
| --- | --- | --- | --- |
|  |  |  |  |
| EcaMb1_{Eca} | PseMb1_{Pse} | 0.2732 | 0.0507 |
| EcaMb2a_{Eca} | PseMb2b_{Pse} | 0.4417 | 0.0814 |
| EcaMb2b_{Eca} | PseMb2a_{Pse} | 0.4337 | 0.0785 |
| EcaMb3_{Eca} | PseMb3_{Pse} | 0.0533 | 0.0188 |
| EcaMb4a_{Eca} | PseMb4c_{Pse} | 0.1189 | 0.0327 |
| EcaMb4b_{Eca} | PseMb4c_{Pse} | 0.1550 | 0.0372 |
| EcaMb5_{Eca} | PseMb5a_{Pse} | 0.2847 | 0.0575 |
| EcaMb7a_{Eca} | PseMb7b_{Pse} | 0.2295 | 0.0496 |
